# Supplementary material for: FFAR4-mediated IL-6 release from islet macrophages promotes insulin secretion and is compromised in type-2 diabetes
Source: Nat Commun. 2025 Apr 10;16:3422. doi: 10.1038/s41467-025-58706-5 (PMC11986018; doi:10.1038/s41467-025-58706-5)

Supplementary Information

of manuscript

FFAR4-mediated IL-6 release from islet macrophages promotes insulin secretion and is compromised in type-2 diabetes

Xinyi Chen, Jingchen Shao, Isabell Brandenburger, Weikun Qian, Lisa Hahnefeld, Rémy Bonnavion, Haaglim Cho, ShengPeng Wang, Juan Hidalgo, Nina Wettschureck, Gerd Geisslinger, Robert Gurke, Zheng Wang, Stefan Offermanns

Supplemental Figures

Suppl. Figure 1

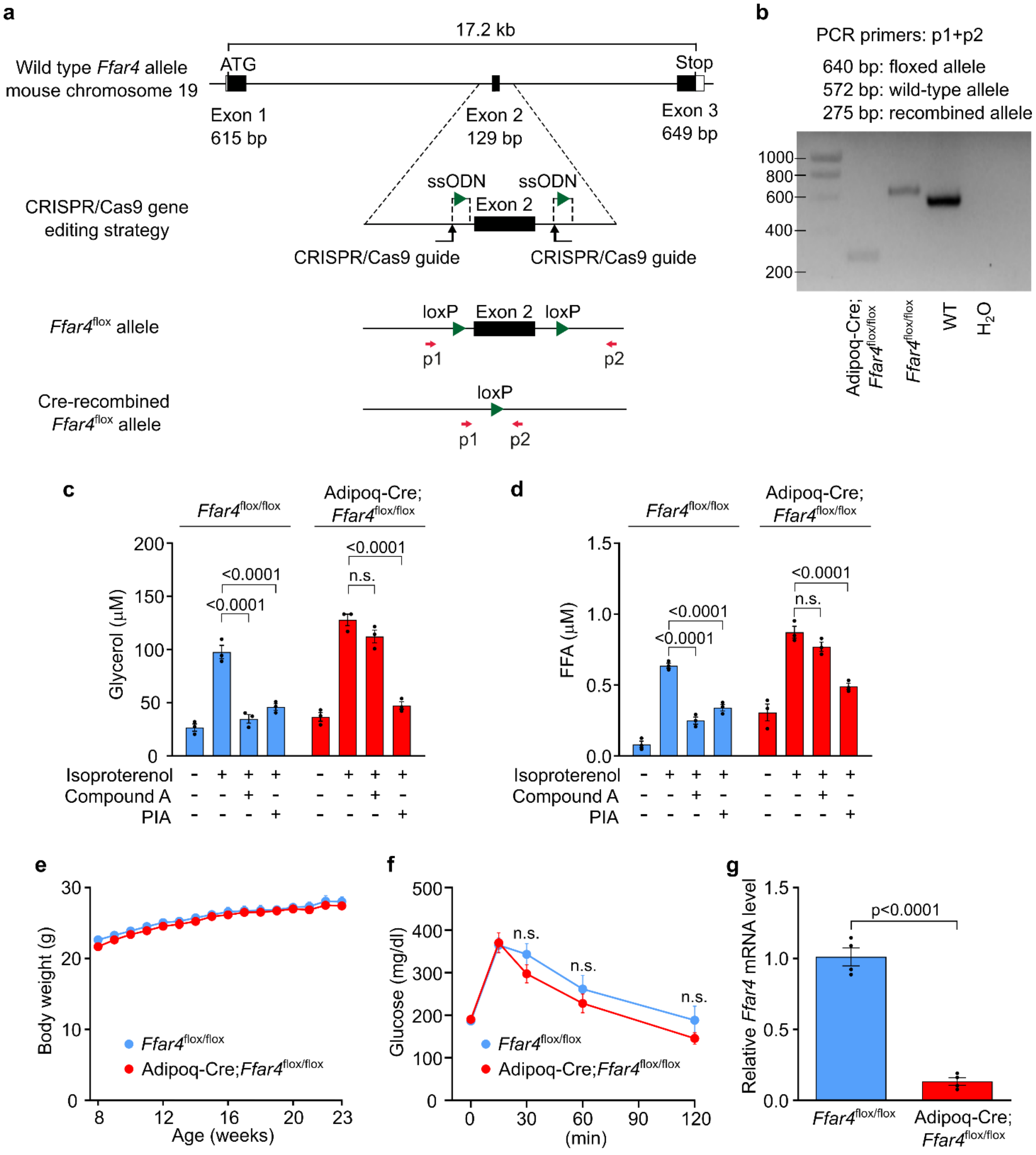

Suppl. Figure 1. Generation of *Ffar4*$^{flox/flox}$ mice. a, Targeting scheme for generating *Ffar4*$^{flox/flox}$ mice using CRISPR/Cas9 gene editing. SsODN, single-stranded oligo donor; p1 and p2, sites corresponding to primers used for genotyping. **b**, Determination of recombination efficiency using genomic DNA of white adipocytes isolated from Adipoq-Cre;*Ffar4*$^{flox/flox}$, *Ffar4*$^{flox/flox}$ or wild-type mice using primer pair p1 and p2. Size of amplified DNAs: 275 bp (recombined allele), 572 bp (wild-type allele), 640 bp (floxed allele). **c** and

d, Release of glycerol (c) or free fatty acids (d) from mature white adipocytes isolated from *Ffar4*$^{flox/flox}$ (control; n=3) and Adipoq-Cre;*Ffar4*$^{flox/flox}$ (n=3) mice. Lipolysis was induced by 100 nM isoproterenol, and the FFAR4 agonist Compound A or the adenosine A1 receptor agonist (R)-N6-(2-Phenylisopropyl) adenosine (PIA) were given at 1 and 10 µM, respectively. PIA served as a positive control for lipolysis inhibition. **e**, Body weight of *Ffar4*$^{flox/flox}$ (control; n=11) and Adipoq-Cre;*Ffar4*$^{flox/flox}$ (n=9) mice fed with normal-chow diet. **f**, Blood glucose levels at the indicated time points after i.p. injection of 2 g/kg glucose in *Ffar4*$^{flox/flox}$ (n=11) and Adipoq-Cre;*Ffar4*$^{flox/flox}$ (n=10) mice. **g**, Recombination efficiency shown as the *Ffar4* mRNA levels in purified white adipocytes isolated from *Ffar4*$^{flox/flox}$ (n=4) and Adipoq-Cre;*Ffar4*$^{flox/flox}$ mice (n=4). Data were normalized to GAPDH, and the control (*Ffar4*$^{flox/flox}$) was set as 1. Shown are mean values ± SEM; p-values are given in the figure; n.s.: not significant (Bonferroni's two-way ANOVA test (c-f) or unpaired parametric Student's *t*-test (g)).

Suppl. Figure 2

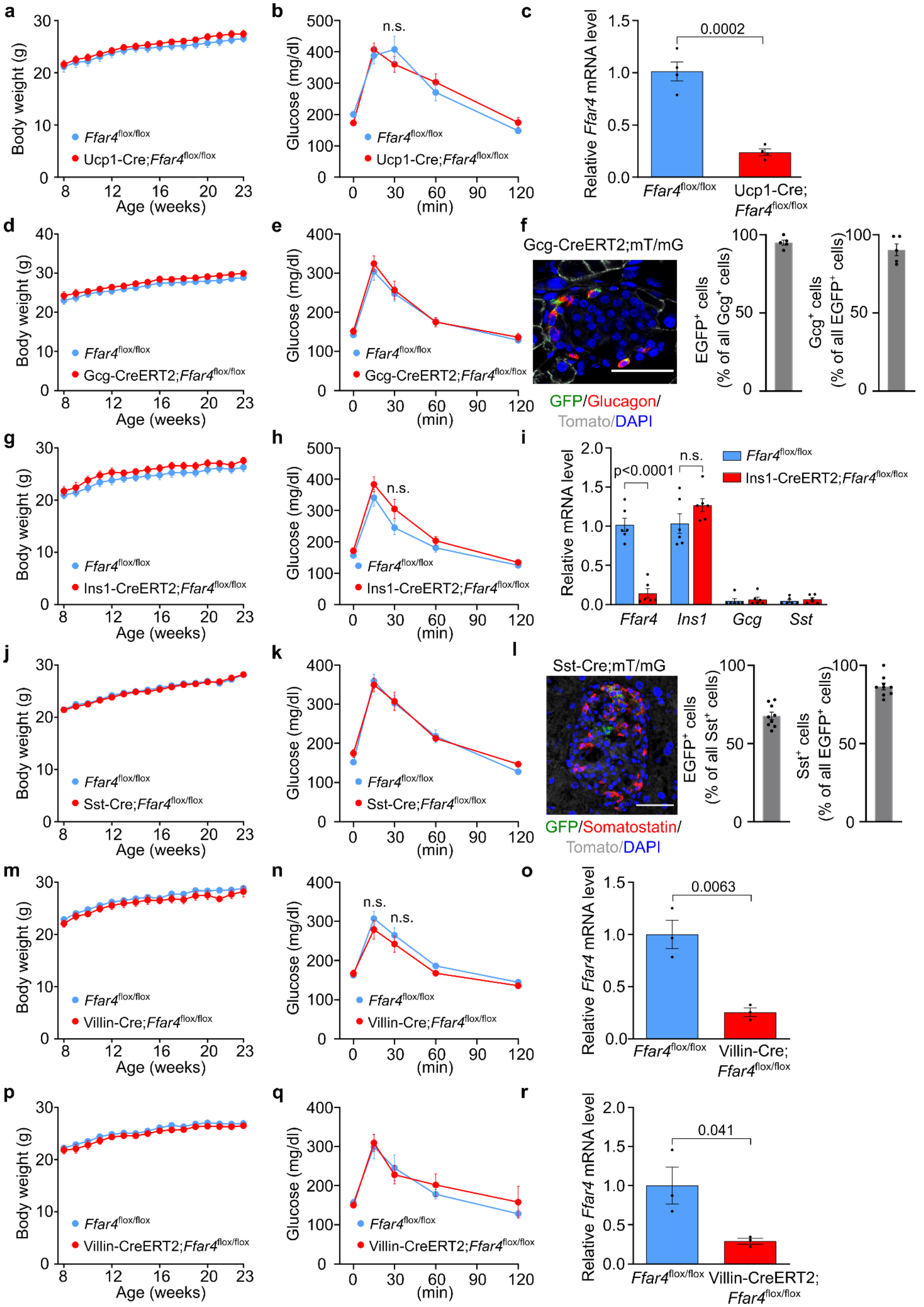

Suppl. Figure 2. Analysis of tissue-specific FFAR4 knockout mice. a, Body weight of *Ffar4*$^{flox/flox}$ (n=6) and Ucp1-Cre;*Ffar4*$^{flox/flox}$ (n=11) mice fed with normal-chow diet. **b**, Blood glucose levels at the indicated time points after i.p. injection of 2 g/kg glucose in *Ffar4*$^{flox/flox}$ (n=6) and Ucp1-Cre;*Ffar4*$^{flox/flox}$ (n=11) mice. **c**, Recombination efficiency shown as the *Ffar4* mRNA levels in purified brown adipocytes harvested from *Ffar4*$^{flox/flox}$ (n=4) and Ucp1-Cre;*Ffar4*$^{flox/flox}$ mice (n=4). Data were normalized to GAPDH and were expressed relative to the average level in brown adipocytes from control (*Ffar4*$^{flox/flox}$) animals. **d**, Body weight of *Ffar4*$^{flox/flox}$ (n=12) and Gcg-CreERT2;*Ffar4*$^{flox/flox}$ (n=10) mice fed with normal-chow diet. **e**, Blood glucose levels at the indicated time points after i.p. injection of 2 g/kg glucose in *Ffar4*$^{flox/flox}$ (n=12) and Gcg-CreERT2;*Ffar4*$^{flox/flox}$ (n=10) mice. **f**, Representative image of pancreatic islets from Gcg-CreERT2;mT/mG mice counterstained with DAPI and anti-glucagon (islet α-cells) antibodies. The bar diagram shows the percentage of EGFP-positive cells of all glucagon (Gcg)-positives cells (left) and the percentage of glucagon-positive cells of all EGFP-positive cells (right). **g**, Body weight of *Ffar4*$^{flox/flox}$ (n=11) and Ins1-CreERT2;*Ffar4*$^{flox/flox}$ (n=10) mice fed with normal-chow diet. **h**, Blood glucose levels at the indicated time points after i.p. injection of 2 g/kg glucose in *Ffar4*$^{flox/flox}$ (n=11) and Ins1-CreERT2;*Ffar4*$^{flox/flox}$ (n=10) mice. **i**, Recombination efficiency shown as the *Ffar4* mRNA levels in sorted islet β-cells isolated from *Ffar4*$^{flox/flox}$ (n=6) and Ins1-CreERT2;*Ffar4*$^{flox/flox}$ mice (n=6). *Ins1*, insulin; *Gcg*, glucagon; *Sst*, somatostatin. Data were normalized to GAPDH and were expressed relative to the average level in the control (*Ffar4*$^{flox/flox}$). **j**, Body weight of *Ffar4*$^{flox/flox}$ (n=11) and Sst-Cre;*Ffar4*$^{flox/flox}$ (n=11) mice fed with normal-chow diet. **k**, Blood glucose levels at the indicated time points after i.p. injection of 2 g/kg glucose in *Ffar4*$^{flox/flox}$ (n=11) and Sst-Cre;*Ffar4*$^{flox/flox}$ (n=11) mice. **l**, Representative image of pancreatic islets from Sst-Cre;mT/mG mice counterstained with DAPI and anti-somatostatin (islet δ-cells) antibodies. The bar diagram shows the percentage of EGFP-positive cells of all somatostatin (Sst)-positive cells (left) and the percentage of Sst-positive cells of all EGFP-positive cells (right). **m**, Body weight of *Ffar4*$^{flox/flox}$ (n=12) and Villin-Cre;*Ffar4*$^{flox/flox}$ (n=10) mice fed with normal-chow diet. **n**, Blood glucose levels at the indicated time points after i.p. injection of 2 g/kg glucose in *Ffar4*$^{flox/flox}$ (n=11) and Villin-Cre;*Ffar4*$^{flox/flox}$ (n=10) mice. **o**, Recombination efficiency shown as the *Ffar4* mRNA levels in sorted intestinal epithelial cells isolated from *Ffar4*$^{flox/flox}$ (n=3) and Villin-Cre;*Ffar4*$^{flox/flox}$ mice (n=3). Data were normalized to GAPDH and were expressed relative to the average level in intestinal epithelial cells from control (*Ffar4*$^{flox/flox}$) animals. **p**, Body weight of *Ffar4*$^{flox/flox}$ (n=10) and induced Villin-CreERT2;*Ffar4*$^{flox/flox}$ (n=12) mice fed with normal-chow diet. **q**, Blood glucose levels at the indicated time points

after i.p. injection of 2 g/kg glucose in *Ffar4*$^{flox/flox}$ (n=10) and induced Villin-CreERT2;*Ffar4*$^{flox/flox}$ (n=11) mice. **r**, Recombination efficiency shown as the *Ffar4* mRNA levels in sorted intestinal epithelial cells isolated from *Ffar4*$^{flox/flox}$ (n=3) and induced Villin-CreERT2;*Ffar4*$^{flox/flox}$ mice (n=3). Data were normalized to GAPDH and were expressed relative to the average level in intestinal epithelial cells from control (*Ffar4*$^{flox/flox}$) animals. Bar length: 50 µm. Shown are mean values ± SEM; p-values are shown in the figure; n.s.: not significant (Bonferroni's two-way ANOVA test (a, b, d, e, g, h, j, k, m, n, p, q) or unpaired parametric Student's *t*-test (c, o, r), or multiple unpaired *t*-test (i)).

Suppl. Figure 3

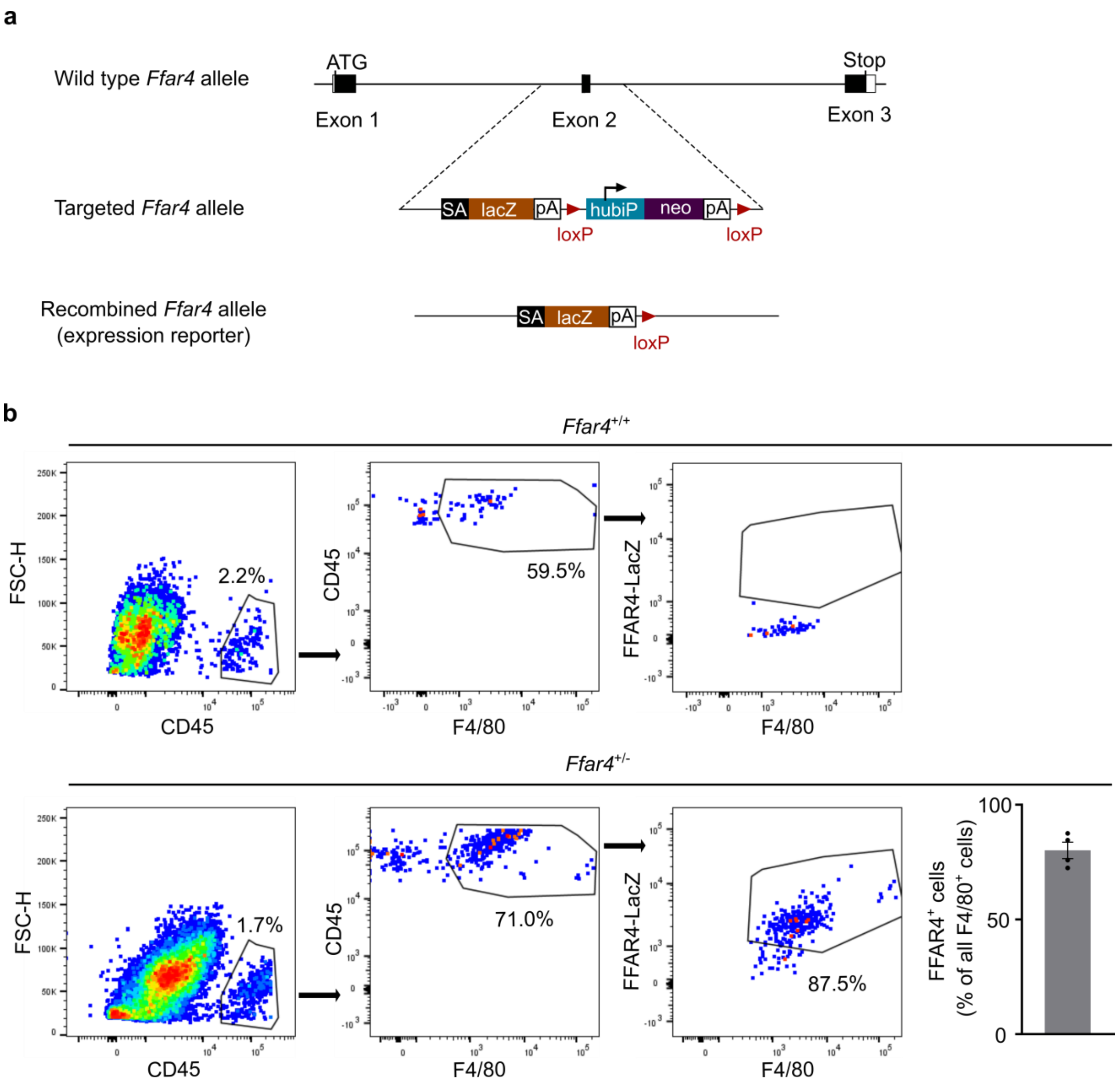

Suppl. Figure 3. *Ffar4* is expressed by islet macrophages. a, Schematic of the targeted allele of *Ffar4* functioning as an expression reporter obtained from the UC Davis KOMP Repository Knockout Mouse Project. In this *Ffar4* allele, the *Ffar4* gene, including exons 1 to 3, was disrupted by gene targeting with a vector (ZEN-UB1) containing a *lacZ* reporter preceded by a splice acceptor sequence (SA) and a selection cassette (neomycin; neo) driven by the promoter from the human ubiquitin C gene (hubiP) and flanked with 2 loxP sites. pA: polyadenylation signal. In this *Ffar4* allele, expression of *lacZ* is driven by the *Ffar4* promoter allowing *Ffar4*-expressing cells to be visualized by β-galactosidase activity. **b**, Expression of FFAR4 in islet macrophages analyzed by FACS using islet cells from *Ffar4*$^{+/+}$ (control) mice and *Ffar4*$^{+/-}$ mice expressing β-galactosidase under the control of the *Ffar4* promoter. Cells were stained with SPiDER-βGal (FFAR4-lacZ) and an anti-F4/80

antibody (macrophages). The bar diagram shows the percentage of FFAR4-positive cells of all F4/80-positive (macrophage) cells. Shown are mean values ± SEM.

Suppl. Figure 4

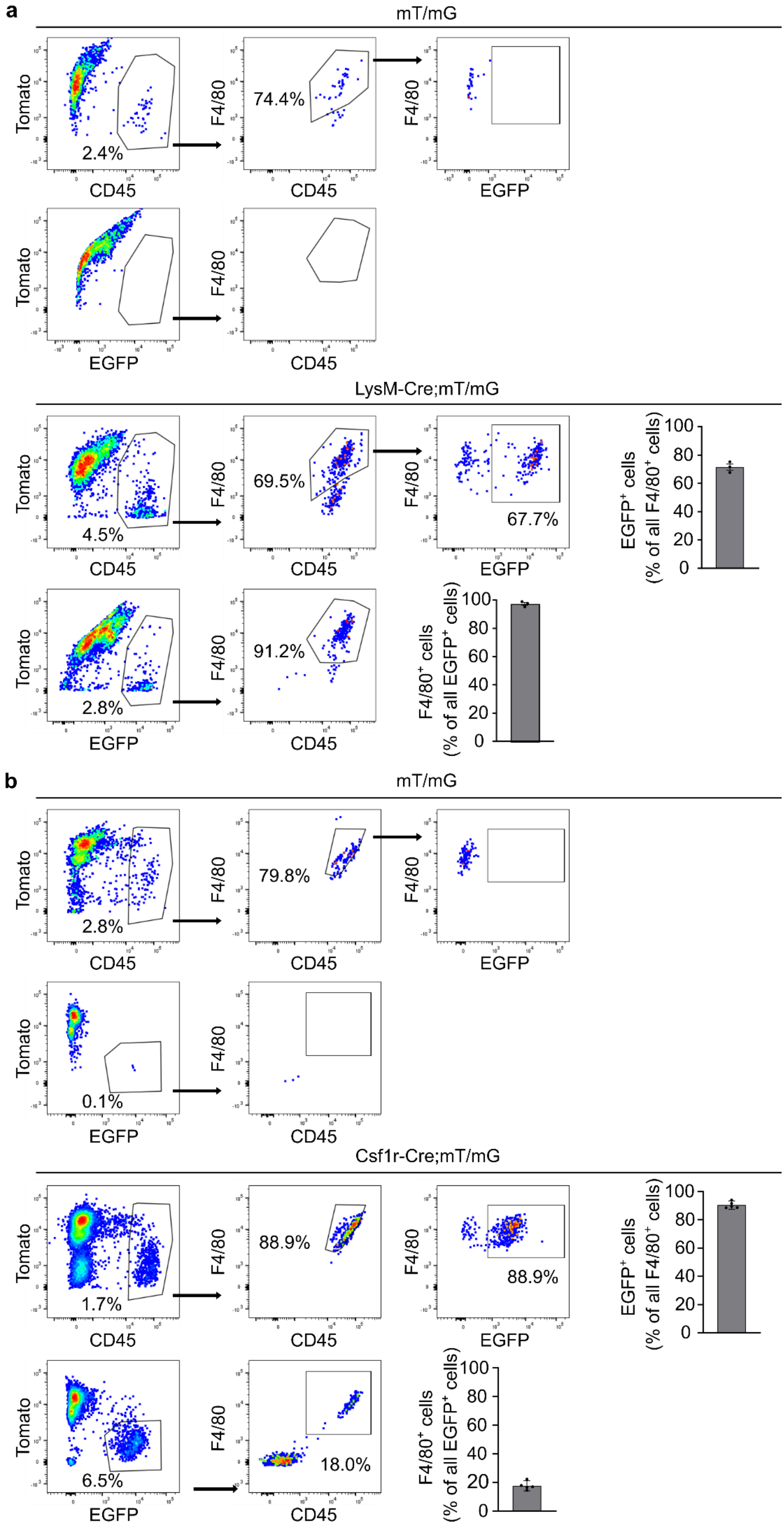

Suppl. Figure 4. Recombination efficiency and specificity of LysM-Cre and Csf1r-Cre mice. a and **b**, Determination of recombination efficiency and specificity of islet macrophages isolated from mTmG, LysM-Cre;mT/mG (a, n=3) and Csf1r-Cre;mT/mG (b, n=5) mice. Analysis was performed using FACS. Cells were stained with DAPI, anti-CD45 (immune cell) and anti-F4/80 (macrophage) antibodies. The bar diagram shows the percentage of EGFP-positive cells (Cre-recombined cells) of all F4/80-positive cells (macrophages; a and b, upper part) and the percentage of F4/80-positive cells (macrophages) of all EGFP-positive cells (Cre-recombined cells; a and b, lower part).

Suppl. Figure 5

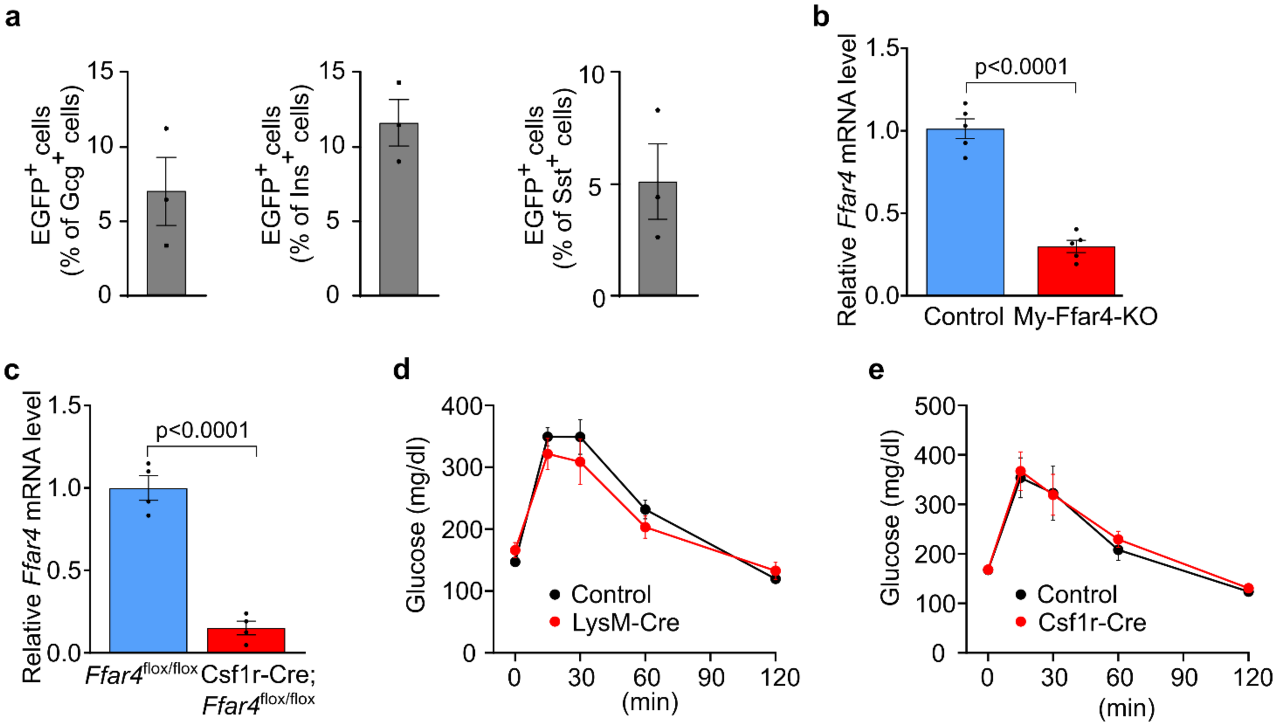

Suppl. Figure 5. Recombination efficiency and specificity of LysM-Cre and Csf1r-Cre mice. a, Sections through islets from Csf1r-Cre;mT/mG mice counterstained with DAPI and anti-glucagon (islet α-cells), anti-insulin (islet β-cells) or anti-somatostatin (islet δ-cells) antibodies were analyzed. Shown is the percentage of GFP-positive recombined cells of all glucagon-positive (islet α-cells), insulin-positive (islet β-cells) or somatostatin-positive (islet δ-cells) cells. **b** and **c,** Recombination efficiency. *Ffar4* mRNA levels in islet macrophages isolated from control (b, n=5) or *Ffar4*$^{flox/flox}$ mice (c, n=4) and My-Ffar4-KO (b, n=5) or Csf1r-Cre;*Ffar4*$^{flox/flox}$ mice (c, n=4). All data were normalized to GAPDH, and controls were set as 1. **d** and **e,** Blood glucose levels at the indicated time points after i.p. injection of 2 g/kg glucose in control (d, n=11; or e, n=6) and LysM-Cre (n=8) or Csf1r-Cre (n=7) mice. Shown are mean values ± SEM; unpaired parametric Student's *t*-test (b, c) or Bonferroni's two-way ANOVA test (d, e).

Suppl. Figure 6

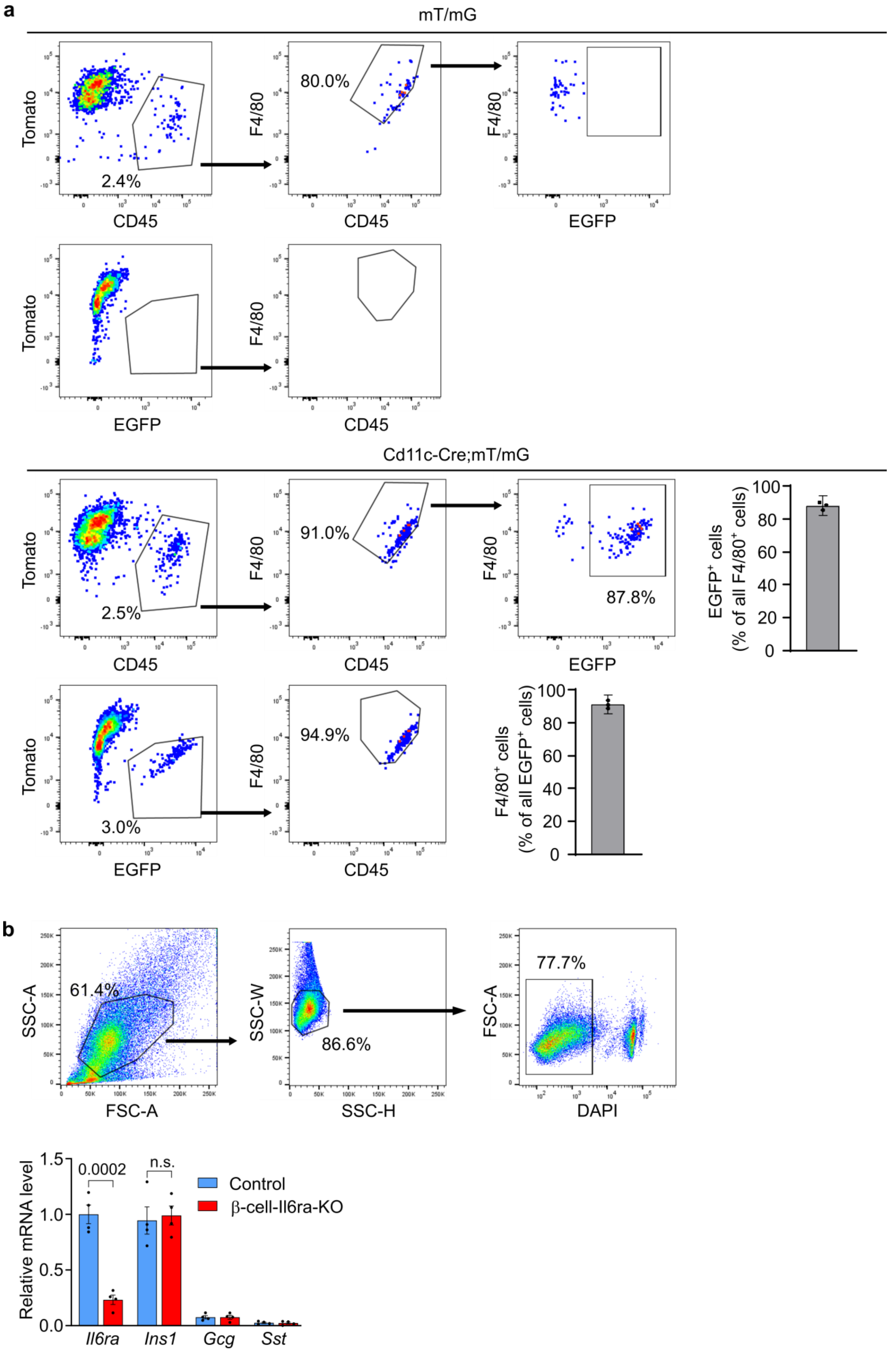

Suppl. Figure 6. Recombination efficiency and specificity of Cd11c-Cre mice and recombination efficiency of β-cell-Il6ra-KO mice. a, Determination of recombination efficiency and specificity of islet macrophages isolated from Cd11c-Cre;mT/mG mice. Analysis was performed using FACS. Cells were stained with DAPI, anti-CD45 and anti-F4/80 antibodies. The bar diagram shows the percentage of EGFP-positive cells of all F4/80-positive cells and the percentage of F4/80-positive cells of all EGFP-positive cells. **b**, Determination of recombination efficiency in β-cell-Il6ra-KO mice. Upper panel: Gating strategy of islet β-cell sorting. Cells were stained with DAPI to exclude dead cells. The analysis was performed by FACS. Lower panel: mRNA was harvested from sorted β-cells, and RT-PCR was performed. Purity of isolated β-cells was determined by testing expression of cell type specific genes (*Ins1*, insulin; *Gcg*, glucagon; *Sst*, somatostatin), and recombination efficiency was analyzed by determining *Il6ra* mRNA levels in islet β-cells isolated from *Il6ra*$^{flox/flox}$ (Control, n=4) and Ins1-CreERT2;*Il6ra*$^{flox/flox}$ (β-cell-Il6ra-KO, n=4) mice. Data were normalized to GAPDH and were expressed relative to the average level in the control. Shown are mean values ± SEM; multiple unpaired *t*-test (b)

Supplemental Tables

Suppl. Table 1. List of analyzed bioactive lipids

Detected in islet supernatants:

Lipid	WT (Average±SD), Arbitrary unit	WT+TUG-891 (Average±SD), Arbitrary unit	P value
FA 20:4	0.010±0.016	0.000	n.s.
FA 22:6	0.004±0.008	0.000	n.s.
LPE 16:0	0.024±0.033	0.000	n.s.
LPE 18:0	0.042±0.047	0.000	n.s.
LPE 20:4	0.052±0.082	0.083±0.036	n.s.
PE 38:4	0.010±0.017	0.021±0.014	n.s.
PI 38:4	0.008±0.016	0.012±0.015	n.s.
LPC 16:0	0.011±0.021	0.000	n.s.

Not detected in islet supernatants:

AHexCer 56:1;O3	AHexCer 58:1;O3	AHexCer 58:2;O3	AHexCer 60:2;O3	CAR 10:0
CAR 10:1	CAR 12:0	CAR 12:1	CAR 13:1	CAR 14:0
CAR 14:1	CAR 16:0	CAR 16:1	CAR 18:0	CAR 18:1
CAR 18:2	CAR 20:0	CAR 20:1	CAR 20:2	CAR 20:4
CAR 22:1	CAR 22:2	CAR 8:0	CE 18:1-d7	Cer 18:0;O2/16:0
Cer 18:0;O2/22:0	Cer 18:0;O2/24:0	Cer 18:0;O2/24:1	Cer 18:1;O2/16:0	Cer 18:1;O2/16:0-d7
Cer 18:1;O2/18:0	Cer 18:1;O2/20:0	Cer 18:1;O2/21:0	Cer 18:1;O2/22:0	Cer 18:1;O2/23:0
Cer 18:1;O2/24:0	Cer 18:1;O2/24:1	Cer 18:1;O2/26:0	Cer 18:1;O2/36:10	Cer 18:1;O2/38:10
Cer 18:2;O2/16:0	Cer 18:2;O2/22:0	Cer 18:2;O2/23:0	Cer 18:2;O2/24:1	Cholesterol-d7
DG 15:0/18:1-d7	DG 30:1	DG 31:0	DG 31:1	DG 32:0
DG 32:1	DG 32:2	DG 33:0	DG 33:1	DG 34:0
DG 34:1	DG 34:2	DG 34:3	DG 35:1	DG 35:2
DG 36:0	DG 36:1	DG 36:2	DG 36:3	DG 36:4
DG 36:5	DG 38:1	DG 38:2	DG 38:3	DG 38:4
DG 38:5	DG 38:6	DG 40:6	DG 40:7	DG 40:8
DG 44:8	Hex2Cer 18:1;O2/16:0	Hex2Cer 18:1;O2/24:0	Hex2Cer 18:1;O2/24:1	HexCer 18:0;O2/18:0
HexCer 18:0;O2/22:0	HexCer 18:0;O2/22:0;O	HexCer 18:0;O2/23:0	HexCer 18:0;O2/24:0	HexCer 18:0;O2/24:0;O

HexCer 18:1;O2/18:0	HexCer 18:1;O2/18:0;O	HexCer 18:1;O2/20:0	HexCer 18:1;O2/20:0;O	HexCer 18:1;O2/21:0;O
HexCer 18:1;O2/22:0	HexCer 18:1;O2/22:0;O	HexCer 18:1;O2/22:1	HexCer 18:1;O2/22:1;O	HexCer 18:1;O2/23:0;O
HexCer 18:1;O2/23:1	HexCer 18:1;O2/23:1;O	HexCer 18:1;O2/24:0	HexCer 18:1;O2/24:0;O	HexCer 18:1;O2/24:1
HexCer 18:1;O2/24:1;O	HexCer 18:1;O2/24:2	HexCer 18:1;O2/24:2;O	HexCer 18:1;O2/25:0;O	HexCer 18:1;O2/25:1
HexCer 18:1;O2/25:1;O	HexCer 18:1;O2/40:2;O	HexCer 18:1;O2/42:2;O	HexCer 18:2;O2/24:0	LacCer 18:1;O2/17:0
LPC 14:0	LPC 15:0	LPC 16:1	LPC 17:0	LPC 18:0
LPC 18:1	LPC 18:1-d7	LPC 18:2	LPC 18:3	LPC 19:0
LPC 19:1	LPC 20:0	LPC 20:1	LPC 20:2	LPC 20:3
LPC 20:4	LPC 20:5	LPC 22:0	LPC 22:4	LPC 22:5
LPC 22:6	LPC 24:0	LPC 24:1	LPC O-16:0-d4	LPC O-24:1
LPE 18:1	LPG 17:1	LPI 17:1	PC 15:0/18:1-d7	PC 25:0
PC 28:0	PC 28:1	PC 29:0	PC 30:0	PC 30:1
PC 30:2	PC 31:0	PC 31:1	PC 31:2	PC 32:0
PC 32:1	PC 32:2	PC 33:0	PC 33:1	PC 33:2
PC 33:3	PC 34:0	PC 34:0;O	PC 34:1	PC 34:2
PC 34:3	PC 34:4	PC 34:5	PC 35:0	PC 35:1
PC 35:2	PC 35:3	PC 35:4	PC 36:0	PC 36:1
PC 36:2	PC 36:3	PC 36:4	PC 36:4;O	PC 36:5
PC 36:6	PC 37:1	PC 37:2	PC 37:3	PC 37:4
PC 37:6	PC 38:0	PC 38:1	PC 38:2	PC 38:3
PC 38:4	PC 38:5	PC 38:6	PC 38:7	PC 39:1
PC 39:2	PC 39:3	PC 39:4	PC 39:5	PC 39:6
PC 39:7	PC 40:0	PC 40:1	PC 40:2	PC 40:4
PC 40:6	PC 40:7	PC 40:8	PC 41:1	PC 41:2
PC 41:6	PC 42:1	PC 42:10	PC 42:2	PC 42:4
PC 42:5	PC 42:6	PC 42:7	PC 42:8	PC 42:9
PC 43:1	PC 43:2	PC 44:1	PC 44:10	PC 44:11
PC 44:12	PC 44:2	PC 44:4	PC 44:5	PC 44:8

PC O-16:0_16:0	PC O-16:0_18:2	PC O-16:0_20:4	PC O-16:1_16:0	PC O-16:1_18:1
PC O-16:1_18:2	PC O-16:1_20:4	PC O-18:0/18:1-d9	PC O-18:0_20:4	PC O-18:1_18:1
PC O-18:1_18:2	PC O-18:1_20:4	PC O-30:0	PC O-30:1	PC O-31:0
PC O-31:1	PC O-32:2	PC O-33:0	PC O-33:1	PC O-34:0
PC O-34:1	PC O-34:4	PC O-36:0	PC O-36:1	PC O-36:6
PC O-37:4	PC O-37:6	PC O-38:1	PC O-38:10	PC O-38:2_1
PC O-38:2_2	PC O-38:3	PC O-38:6	PC O-38:7	PC O-38:9
PC O-39:1	PC O-39:6	PC O-39:8	PC O-40:10	PC O-40:11
PC O-40:2	PC O-40:4	PC O-40:5	PC O-40:6	PC O-40:7
PC O-40:8	PC O-40:9	PC O-42:10	PC O-42:3	PC O-42:4
PC O-42:5	PC O-42:6	PC O-42:7	PC O-44:5	PC O-44:6
PC O-44:7	PC O-46:7	PE 15:0/18:1-d7	PG 15:0/18:1-d7	PI 15:0/18:1-d7
PS 15:0/18:1-d7	SE 27:1/14:0	SE 27:1/15:0	SE 27:1/16:2	SE 27:1/17:0
SE 27:1/17:1	SE 27:1/18:1	SE 27:1/18:2	SE 27:1/18:3	SE 27:1/20:1
SE 27:1/20:2	SE 27:1/20:3	SE 27:1/20:4	SE 27:1/20:5	SE 27:1/22:4
SE 27:1/22:5	SE 27:1/22:6	SE 27:1/24:6	SM 18:1;O2/18:1-d9	SM 30:0;O2
SM 30:1;O2	SM 32:0;O2	SM 32:1;O2	SM 32:2;O2	SM 33:1;O2
SM 33:2;O2	SM 34:0;O2	SM 34:1;O2	SM 34:1;O2	SM 34:2;O2
SM 35:0;O2	SM 35:1;O2	SM 35:2;O2	SM 36:0;O2	SM 36:1;O2
SM 36:2;O2	SM 36:3;O2	SM 37:1;O2	SM 37:2;O2	SM 38:0;O2
SM 38:1;O2	SM 38:2;O2	SM 38:3;O2	SM 39:1;O2	SM 39:2;O2
SM 40:0;O2	SM 40:1;O2	SM 40:2;O2	SM 40:3;O2	SM 40:4;O2
SM 41:0;O2	SM 41:1;O2	SM 41:2;O2	SM 41:3;O2	SM 42:0;O2
SM 42:0;O3	SM 42:1;O2	SM 42:1;O3	SM 42:2;O2	SM 42:3;O2
SM 42:4;O2	SM 42:5;O2	SM 43:1;O2	SM 43:2;O2	SM 43:3;O2
SM 44:1;O2	SM 44:2;O2	SM 44:3;O2	ST 27:1;O	TG 14:0/16:1/14:0-d5
TG 15:0/18:1-d7/15:0	TG 20:0/20:1/20:0-d5	TG 24:0	TG 26:0	TG 28:0
TG 34:0	TG 36:1	TG 42:0	TG 42:1	TG 42:2

TG 43:0	TG 44:0	TG 44:1	TG 44:2	TG 44:3
TG 45:0	TG 45:1	TG 46:0	TG 46:1	TG 46:2
TG 46:3	TG 47:0	TG 47:1	TG 47:2	TG 48:0
TG 48:1	TG 48:2	TG 48:3	TG 48:4	TG 49:0
TG 49:1	TG 49:2	TG 49:3	TG 49:4	TG 50:0
TG 50:1	TG 50:2	TG 50:3	TG 50:4	TG 50:5
TG 51:0	TG 51:1	TG 51:2	TG 51:3	TG 51:4
TG 52:0	TG 52:1	TG 52:2	TG 52:3	TG 52:4
TG 52:5	TG 52:6	TG 52:7	TG 53:0	TG 53:1
TG 53:2	TG 53:3	TG 53:4	TG 53:5	TG 54:0
TG 54:1	TG 54:2	TG 54:3	TG 54:4	TG 54:5
TG 54:6	TG 54:7	TG 55:0	TG 55:1	TG 55:2
TG 55:3	TG 55:4	TG 55:5	TG 55:6	TG 56:0
TG 56:1	TG 56:2	TG 56:3	TG 56:4	TG 56:5
TG 56:6	TG 56:7	TG 56:8	TG 56:9	TG 57:1
TG 57:2	TG 57:3	TG 57:4	TG 58:1	TG 58:10
TG 58:11	TG 58:2	TG 58:3	TG 58:4	TG 58:5
TG 58:6	TG 58:7	TG 58:8	TG 58:9	TG 59:2
TG 59:3	TG 59:4	TG 60:10	TG 60:11	TG 60:12
TG 60:2	TG 60:3	TG 60:4	TG 60:5	TG 60:6
TG 60:7	TG 60:8	TG 62:2	TG 63:3	TG 70:3
TG 72:3	TG O-50:1	TG O-50:2	TG O-50:3	TG O-52:3
Tributyl citrate	Ubiquinone-10	Arachidonic acid-d8	Cer 18:1;O2/16:0-d7	FA 14:0
FA 15:0	FA 16:0	FA 16:1	FA 17:0	FA 17:1
FA 18:0	FA 18:1	FA 18:2	FA 18:3	FA 18:4
FA 20:1	FA 20:2	FA 20:5	FA 22:4	FA 24:0
FA 24:1	LPC 18:1-d7	LPC O-16:0	LPC O-16:0-d4	LPC O-16:1
LPC O-17:1	LPC O-18:0	LPC O-18:1	LPC O-18:2	LPC O-20:0

LPE 16:1	LPE 17:0	LPE 17:2	LPE 18:1	LPE 18:1-d7
LPE 18:2	LPE 18:3	LPE 20:0	LPE 20:1	LPE 20:2
LPE 20:3	LPE 20:5	LPE 22:0	LPE 22:3	LPE 22:4
LPE 22:5	LPE 22:6	LPE 24:0	LPE O-16:1	LPE O-18:1
LPE O-18:2	LPE O-20:1	LPG 16:0	LPG 17:1	LPG 18:0
LPG 18:1	LPG 18:2	LPI 16:0	LPI 17:1	LPI 18:0
LPI 18:1	LPI 18:2	LPI 20:3	LPI 20:4	LPS 18:0
LPS 18:1	LPS 20:4	LPS 22:6	NeuAcHex2Cer 34:1;O2	PA 34:1
PA 36:1	PA 36:2	PC 15:0/18:1-d7	PC 34:1;O	PC 34:2;O2
PC 36:1;O	PC 36:2;O	PC 36:3;O2	PC 36:3;O	PC 38:5;O
PC 18:0/18:1;O-d9	PE 15:0/18:1-d7	PE 32:0	PE 32:1	PE 34:1
PE 34:2	PE 34:3	PE 35:2	PE 36:1	PE 36:2
PE 36:3	PE 36:4	PE 36:5	PE 37:4	PE 38:2
PE 38:6	PE 40:1	PE 40:3	PE 40:4	PE 40:5
PE 40:6	PE 40:7	PE 40:8	PE 41:1	PE 42:1
PE 42:10	PE 42:7	PE 42:8	PE 44:10	PE 44:11
PE O-16:1_16:0	PE O-16:1_18:1	PE O-16:1_18:2	PE O-16:1_20:4	PE O-16:1_22:6
PE O-18:1_16:0	PE O-18:1_18:1	PE O-18:1_18:2	PE O-18:1_20:4	PE O-18:1_22:6
PE O-18:2_14:0	PE O-18:2_18:2	PE O-18:2_20:4	PE O-35:2	PE O-35:3
PE O-36:1	PE O-36:6	PE O-38:1	PE O-38:2	PE O-38:3
PE O-38:4	PE O-40:2	PE O-40:3	PE O-40:5	PE O-40:6
PE O-40:8	PE O-42:3	PE O-42:5	PE O-42:6	PG 15:0/18:1-d7
PG 30:0	PG 32:0	PG 32:1	PG 34:1	PG 34:2
PG 36:1	PG 36:2	PG 36:3	PG 36:4	PG 38:4
PG 38:5	PG 38:6	PG 40:6	PG 40:7	PG 40:8
PI 15:0/18:1-d7	PI 32:0	PI 32:1	PI 33:1	PI 34:0
PI 34:1	PI 34:2	PI 35:1	PI 35:2	PI 36:1
PI 36:2	PI 36:3	PI 36:4	PI 36:5	PI 37:4

PI 38:3	PI 38:5	PI 38:6	PI 40:5	PI 40:6
PI 1O-8:1_20:4	PS 15:0/18:1-d7	PS 34:1	PS 36:1	PS 36:2
PS 36:4	PS 38:1	PS 38:2	PS 38:3	PS 38:4
PS 38:5	PS 38:6	PS 40:1	PS 40:2	PS 40:4
PS 40:5	PS 40:6	PS 40:7	PS 42:1	PS 42:2
PS 44:10	PS 44:12	SHexCer 36:1;O2	SHexCer 36:1;O3	SHexCer 38:1;O2
SHexCer 38:1;O3	SHexCer 40:1;O2	SHexCer 40:1;O3	SHexCer 40:2;O2	SHexCer 41:1;O3
SHexCer 42:0;O2	SHexCer 42:1;O2	SHexCer 42:2;O2	SHexCer 42:2;O3	SHexCer 42:3;O2
SHexCer 44:2;O2	SM 18:1;O2/18:1-d9	ST 19:2;O2;S	ST 19:2;O2;S	ST 27:1;O;S
Thyroxin				

Suppl. Table 2. List of analyzed polar metabolites

Detected in islet supernatants:

Metabolites	WT (Average±SD), AUC, counts/second	WT+TUG-891 (Average±SD), AUC, counts/second	P value
cis-Aconitic acid	1.55E+08±9.76E+07	4.07E+07±1.09E+07	n.s.
Dihexose	9.75E+05±1.13E+06	4.50E+05±5.45E+05	n.s.
Fumaric acid	7.39E+06±4.57E+06	1.25E+06±4.16E+05	n.s.
Hexose	1.45E+09±1.77E+09	8.25E+08±9.74E+08	n.s.
Hippuric acid	1.25E+06±1.50E+06	1.43E+06±1.59E+06	n.s.
Hydroxymethylglutaric acid	1.83E+05±3.65E+05	0	n.s.
Maleic acid	5.72E+06±3.04E+06	1.09E+06±7.62E+05	n.s.
Mannitol / Iditol	1.05E+06±2.10E+06	0	n.s.
N-Acetylalanine	1.00E+06±1.29E+06	4.75E+05±9.50E+05	n.s.
N-Acetylaspartic acid	1.49E+06±1.68E+06	2.44E+06±2.05E+06	n.s.
N-Acetylglutamine	8.89E+06±5.33E+06	1.62E+07±2.79E+06	n.s.
Orotic acid	1.97E+07±1.41E+07	3.77E+07±1.27E+07	n.s.
Oxoproline	2.38E+08±3.65E+08	0	n.s.
Pentose	6.75E+05±1.10E+06	0	n.s.
Pseudouridine	1.98E+06±1.80E+06	1.74E+06±1.33E+06	n.s.
Succinic acid	1.25E+06±2.50E+06	0	n.s.
Uridine	1.09E+07±1.20E+06	1.36E+07±1.69E+06	n.s.
Xanthosine	1.41E+06±1.03E+06	2.75E+03±5.50E+03	n.s.
Xylitol / Arabitol	1.00E+05±2.00E+05	0	n.s.
Acetyllysine	6.35E+05±2.35E+05	2.01E+06±1.53E+06	n.s.
Alanine/Sarcosine	1.76E+07±1.09E+07	8.45E+06±8.68E+06	n.s.
alpha-Aminoadipic acid	1.68E+06±2.25E+06	0	n.s.
Dimethylglycine/Aminobutyric acid	1.93E+07±3.40E+06	1.22E+07±4.17E+06	n.s.
Aminooctanoic acid	3.75E+04±7.50E+04	0	n.s.

Arginine	1.60E+09±2.09E+09	1.05E+09±1.67E+09	n.s.
Asparagine	2.18E+07±2.81E+07	1.68E+07±1.99E+07	n.s.
Aspartic acid	4.75E+06±5.50E+06	6.75E+06±3.20E+06	n.s.
Hydroxyproline	1.40E+07±1.93E+07	9.75E+06±1.32E+07	n.s.
Cystathionine	4.57E+06±1.60E+06	3.32E+06±1.17E+06	n.s.
Cystine	2.75E+08±4.86E+08	3.25E+08±3.95E+08	n.s.
Dimethylarginine	3.76E+06±2.73E+06	5.81E+06±2.65E+06	n.s.
DOPA	1.05E+06±1.84E+06	4.73E+06±5.62E+06	n.s.
Glutamic acid	2.25E+07±2.64E+07	1.80E+07±2.23E+07	n.s.
Glutamine	1.25E+08±1.45E+08	8.25E+07±1.02E+08	n.s.
Glycine	1.40E+07±1.59E+07	1.85E+07±1.83E+07	n.s.
Hydroxybenzaldehyde	3.23E+05±3.77E+05	2.00E+04±4.00E+04	n.s.
Hypoxanthine	2.50E+04±5.00E+04	0	n.s.
Inosine	4.26E+06±4.64E+05	3.49E+06±1.59E+06	n.s.
Leucine/Isoleucine	0	2.75E+08±5.50E+08	n.s.
Lysine	1.10E+08±1.60E+08	1.15E+08±1.45E+08	n.s.
Methionine	3.65E+08±4.52E+08	3.60E+08±4.38E+08	n.s.
N-Acetylarginine	2.98E+06±7.79E+05	8.07E+06±5.13E+06	n.s.
Niacinamide	2.60E+07±4.56E+07	0	n.s.
Ornithine	2.24E+08±1.13E+08	2.93E+08±4.97E+07	n.s.
Phenylalanine	8.75E+07±1.75E+08	1.68E+08±2.67E+08	n.s.
Pipecolic acid	1.13E+07±1.60E+07	1.75E+07±2.10E+07	n.s.
Proline	1.10E+08±1.29E+08	1.15E+08±1.56E+08	n.s.
Pyroglutamic acid	1.65E+07±1.91E+07	1.38E+07±1.63E+07	n.s.
Serine	1.50E+07±2.38E+07	7.50E+05±1.50E+06	n.s.
Threonine	2.33E+07±2.93E+07	1.73E+07±2.17E+07	n.s.
Tyrosine	2.63E+08±3.25E+08	1.48E+08±1.94E+08	n.s.
Uracil	1.98E+06±4.07E+05	2.04E+06±4.46E+05	n.s.
Valine	3.00E+08±4.20E+08	1.95E+08±1.70E+08	n.s.

Xanthine	4.50E+05±9.00E+08	0	n.s.

Not detected in islet supernatants:

Citric acid-13C3	Fructose-13C6	Glutamic acid-d5	Hypoxanthine-13C5	Succinic acid-d4
Acetamidobutanoic acid	Allantoin	alpha-N-Phenylacetylglutamine	Aminohippuric acid	AMP
Ascorbic acid sulfate	BA 24:1;O3;G	BA 24:1;O3;G;S	BA 24:1;O3;T	BA 24:1;O4
BA 24:1;O4;G	BA 24:1;O4;T	BA 24:1;O5	BA 24:1;O5;G	BA 24:1;O5;T
Bilirubin	Citric acid	Cysteic acid	Dihydroxybenzoic acid	Dimethyluric acid
Ethylmalonic acid / Methylsuccinic acid / Glutaric acid	Galacturonic acid	Gluconic acid	Glucuronic acid	Glucuronolactone
Glutathione	Glycerophospho-N-palmitoyl ethanolamine	Gly-Leu	GMP	Guanidoacetic acid
Guanosine	Hexose 6-phosphate	Hydroxymethylglutaric acid	Hydroxyphenyllactic acid	IMP
Indolelactic acid	Indoxyl sulfate	Lactic acid	Malic acid	Malic acid
Mandelic acid / Hydroxyphenylacetic acid		Methoxyhydroxyphenylethyleneglycol sulfate	Methylpentose	Methyluric acid
N-Acetyl-Asp-Glu	N-Acetyl-D-galactosamine 4-sulphate	N-Acetylglutamic acid	N-Acetylneuraminic acid	N-Acetyl-tryptophan
Orotidine	P-Cresol	p-Cresol sulfate	Quinic acid	Ribose 5-phosphate
Saccharopine	S-adenosylhomocysteine	Salicyluric acid	ST 19:2;O2;S	Threonic acid
Trimethyluric acid	UMP	Ureidopropionic acid	Uric acid	Vitamin C

Xanthurenic acid	Carnitine-d9	Glutamic acid-d5	Glycine-d2	Hypoxanthine-13C5
Leucine-d10	Lysine-d9	5-Hydroxylysine	Acetamidobutanoic acid	Adenosine
Anserine	Betaine	CAR 2:0	CAR 3:0	CAR 4:0
CAR 5:0	CAR 6:0	CAR 8:0	CAR DC3:0;2Me	CAR DC5:0
CAR DC5:0;3Me	Carnitine	Carnosine	Choline	Citrulline
Creatine	Creatinine	Cysteine	Cysteine-S-sulfate	Cytidine
Dopamine	Ergothioneine	Guanidinobutanoic acid	Guanidinosuccinic acid	Guanine
Histamine	Histidine	Homoarginine	Homocysteine	Hypotaurine
Indoleacrylic acid	Indolepropionic acid	Kynurenic acid	Kynurenine	Leu-Pro
Methionine sulfoxide	Methyladenosine	Methylhistamine	Methylhistidine	Methylimidazole acetic acid
Methylxanthine	N4-Acetylcytidine	N-6-Trimethyllysine	N-Acetylcarnosine	N-Acetylornithine
N-Acetylserine	O-Acetylserine	Pantothenic acid	Paraxanthine / Theobromine / Theophylline	Phenylethylamine
Phenylglycine	Proline betaine	Serotonin	Taurine	Trigonelline
Trimethylamine N-oxide	Tryptophan	Urocanic acid		

Suppl. Table 3. Parameters of lipidomic and metabolomic analysis

Lipidomics: LC-MS/MS Instrumentation and Analysis

Instrumentation

HPLC:	Thermo Vanquish Horizon
MS:	Thermo Orbitrap Exploris 480
Mode	H-ESI - positive and negative electrospray ionization
Column	Zorbax RRHD Eclipse Plus C8 2.1 x 50 mm, 1.8µm (Agilent)
Pre-column	Zorbax Eclipse Plus C8 2.1 mm, 1.8 µm (Agilent)

Analysis

Data Acquisition	Xcalibur 4.4
Data Evaluation	TraceFinder 5.1
Method	Scan + ddMS^2

LC parameters

Solvents

A	Water + 0,1 % formic acid + 10 mM ammonium formate
B	Acetonitrile:isopropanol (2:3) + 0.1% formic acid

Gradient Elution

Time [min]	A [%]	B [%]	Flowrate [mL/min]
0.00	75.00	25.00	0.300
0.30	75.00	25.00	0.300
1.50	20.00	80.00	0.300
11.00	0.00	100.00	0.300
12.00	0.00	100.00	0.300
12.50	75.00	25.00	0.300
14.00	75.00	25.00	0.300

Injection Volume

Positive Ionization Mode	2 µL
Negative Ionization Mode	5 µL

Positive Ionization

MS parameters

Ion Source

Parameters	Value
Ion Source Type:	H-ESI
Spray Voltage:	Static
Positive Ion (V):	3000
Negative Ion (V):	2500
Gas Mode:	Static
Sheath Gas (Arb):	40
Aux Gas (Arb):	20
Sweep Gas (Arb):	0
Ion Transfer Tube Temp (°C):	260
Vaporizer Temp (°C):	290

MS Global Settings

Parameters	Value
Infusion Mode:	Liquid Chromatography
Expected LC Peak Width (s):	6
Advanced Peak Determination:	TRUE
Mild Trapping:	TRUE
Default Charge State:	1

Negative Ionization

MS parameters

Ion Source

Parameters	Value
Ion Source Type:	H-ESI
Spray Voltage:	Static
Positive Ion (V):	3000
Negative Ion (V):	2500
Gas Mode:	Static
Sheath Gas (Arb):	40
Aux Gas (Arb):	20
Sweep Gas (Arb):	0
Ion Transfer Tube Temp (°C):	260
Vaporizer Temp (°C):	290

MS Global Settings

Parameters	Value
Infusion Mode:	Liquid Chromatography
Expected LC Peak Width (s):	6
Advanced Peak Determination:	TRUE
Mild Trapping:	TRUE
Default Charge State:	1

Enable Xcalibur AcquireX method configurations TRUE
Internal Mass Calibration: RunStart EASY-IC™

Settings for Full Scan
Orbitrap Resolution: 120000
Scan Range (m/z): 180-1500
RF Lens (%): 25.0
AGC Target: Custom
Normalized AGC Target (%): 100.0
Maximum Injection Time Mode: Auto
Microscans: 1.0
Data Type: Profile
Polarity: Positive
Source Fragmentation: Disabled

Filters
Intensity
Filter Type: Intensity Threshold
Intensity Threshold: 5000.0

Dynamic Exclusion
Dynamic Exclusion Mode: Custom
Exclude after n times: 1.0
Exclusion duration (s): 5.0
Mass Tolerance: ppm
Low 10.0
High 10.0
Exclude isotopes: TRUE
Perform dependent scan on single charge state p FALSE

Targeted Mass
Mass List
Mass List Type: m/z
Time Mode: Start/End Time
Include Intensity Threshold: TRUE
Add Mass List Targets Determined by Xcalibur A TRUE

Compound	m/z	t start (min)	t stop (min)	Intensity Threshold
MRFA (Placeholder)	524.265	0.0	14.0	0.0

Mass Tolerance: ppm
Low: 10.0
High: 10.0
Set Collision Energy per Compound: FALSE
Perform dependent scan on most intense ion if n TRUE

Targeted Mass Exclusion
Mass List
Mass List Type: m/z
Time Mode: Start/End Time
Include Intensity Threshold: TRUE
Add Mass List Targets Determined by Xcalibur A TRUE

Compound	m/z	t start (min)	t stop (min)	Intensity Threshold
Mass list created via AcquireX				

Mass Tolerance: ppm
Low: 10.0
High: 10.0

Apex Detection
Desired Apex Window (%): 30.0

Data Dependent
Data Dependent Mode: Cycle Time
Time between Master Scans (sec): 0.6

Settings for ddMS^2

Enable Xcalibur AcquireX method TRUE
Internal Mass Calibration: RunStart EASY-IC™

Settings for Full Scan
Orbitrap Resolution: 120000
Scan Range (m/z): 180-1500
RF Lens (%): 25.0
AGC Target: Custom
Normalized AGC Target (%): 100.0
Maximum Injection Time Mode: Auto
Microscans: 1.0
Data Type: Profile
Polarity: Negative
Source Fragmentation: Disabled

Filters
Intensity
Filter Type: Intensity Threshold
Intensity Threshold: 5000.0

Dynamic Exclusion
Dynamic Exclusion Mode: Custom
Exclude after n times: 1.0
Exclusion duration (s): 5.0
Mass Tolerance: ppm
Low 10.0
High 10.0
Exclude isotopes: TRUE
Perform dependent scan on single FALSE

Targeted Mass
Mass List
Mass List Type: m/z
Time Mode: Start/End Time
Include Intensity Threshold: TRUE
Add Mass List Targets Determined TRUE

Compound	m/z	t start (min)	t stop (min)	Intensity Threshold
MRFA (Placeholder)	524.265	0.0	14.0	0.0

Mass Tolerance: ppm
Low: 10.0
High: 10.0
Set Collision Energy per Compour FALSE
Perform dependent scan on most TRUE

Targeted Mass Exclusion
Mass List
Mass List Type: m/z
Time Mode: Start/End Time
Include Intensity Threshold: TRUE
Add Mass List Targets Determined TRUE

Compound	m/z	t start (min)	t stop (min)	Intensity Threshold
Mass list created via AcquireX				

Mass Tolerance: ppm
Low: 10.0
High: 10.0

Apex Detection
Desired Apex Window (%): 30.0

Data Dependent
Data Dependent Mode: Cycle Time
Time between Master Scans (sec) 0.6

Settings for ddMS^2

Multiplex Ions:	FALSE	Multiplex Ions:	FALSE
Isolation Window (m/z):	1.0	Isolation Window (m/z):	1.0
Isolation Offset:	Off	Isolation Offset:	Off
Collision Energy Mode:	Stepped	Collision Energy Mode:	Stepped
Collision Energy Type:	Normalized	Collision Energy Type:	Normalized
HCD Collision Energies (V):	15,30,50	HCD Collision Energies (V):	15,30,50
Orbitrap Resolution:	15000.0	Orbitrap Resolution:	15000.0
Scan Range Mode:	Auto	Scan Range Mode:	Auto
AGC Target:	Custom	AGC Target:	Custom
Normalized AGC Target (%):	10.0	Normalized AGC Target (%):	10.0
Maximum Injection Time Mode:	Auto	Maximum Injection Time Mode:	Auto
Microscans:	1.0	Microscans:	1.0
Data Type:	Centroid	Data Type:	Centroid

**Setting for AcquireX**	
Algorithm:	Background Exclusion
Description:	Create and use an exclusion list of constant background ions and peaks to reduce background fragmentation in your ID runs.
Used reference sample:	An extraced blank sample was used to create the exclusion lists for positive and negative ionization modes.
Exclusion Override Factor:	3.0
Excluison List Peak Window Extension (s):	6.0

Internal Standards

Isotopically labeled internal standard	Vendor	Category	Concentration of standar	Volume of working soluti	Sample Volume [µL]	Concentration related to sample [ng/mL]
Arachidonic acid-d8	Cayman Chemicals	D	100.0	75.0	10.0	750
CE 18:1-d7	Avanti Polar Lipids	J	5000.0	75.0	10.0	37500
Cer d18:1/16:0-d7	Avanti Polar Lipids	A	20.0	75.0	10.0	150
Cholesterol-d7	Avanti Polar Lipids	K	7506.7	75.0	10.0	56300.25
DG 15:0/18:1-d7	Avanti Polar Lipids	F	300.0	75.0	10.0	2250
LacCer d18:1/17:0	Avanti Polar Lipids	C	60.0	75.0	10.0	450
LPC 18:1-d7	Avanti Polar Lipids	F	300.0	75.0	10.0	2250
LPC O-16:0-d4	Cayman Chemicals	A	20.0	75.0	10.0	150
LPE 18:1-d7	Avanti Polar Lipids	A	20.0	75.0	10.0	150
LPG 17:1	Avanti Polar Lipids	A	20.0	75.0	10.0	150
LPI 17:1	Avanti Polar Lipids	A	20.0	75.0	10.0	150
PC 15:0/18:1-d7	Avanti Polar Lipids	I	2000.0	75.0	10.0	15000
PC O-18:0/18:1-d9	Avanti Polar Lipids	E	200.0	75.0	10.0	1500
PE 15:0/18:1-d7	Avanti Polar Lipids	D	100.0	75.0	10.0	750
PG 15:0/18:1-d7	Avanti Polar Lipids	D	100.0	75.0	10.0	750
PI 15:0/18:1-d7	Avanti Polar Lipids	D	100.0	75.0	10.0	750
PS 15:0/18:1-d7	Avanti Polar Lipids	B	25.1	75.0	10.0	188.25
SM d18:1/18:1-d9	Avanti Polar Lipids	G	400.0	75.0	10.0	3000
TG 14:0/16:1/14:0-d5	Avanti Polar Lipids	H	600.0	75.0	10.0	4500
TG 15:0/18:1-d7/15:0	Avanti Polar Lipids	H	600.0	75.0	10.0	4500
TG 20:0/20:1/20:0-d5	Avanti Polar Lipids	H	600.0	75.0	10.0	4500

*Avanti Polar Lipids Alabaster, AL, USA
*Cayman Chemicals Ann Arbor, MI, USA

Acceptance criteria

The following analytes were excluded from data evaluation:
- Analytes showing a mass error > 5 ppm
- Analytes showing an isotope score < 40 (10 ppm, 90% intensity)
- Analytes without identity confirmation via spectral library matching or reference substance measurements
- Analytes containing only missing values in QC samples and study samples
- Analytes containing > 50% missing values in QC samples and study samples
- Analytes showing > 30% area RSD in QC samples

Acquisition List

Compound Name	Compound Type	Compound Formula	m/z	Polarity	Adduct	Charge State	Retention Time
AHexCer 56:1;O3	Target Compound	C62H119NO10	1038.89068	Positive	M+H	1	8.95
AHexCer 58:1;O3	Target Compound	C64H123NO10	1066.92198	Positive	M+H	1	9.34
AHexCer 58:2;O3	Target Compound	C64H121NO10	1064.90633	Positive	M+H	1	8.97
AHexCer 60:2;O3	Target Compound	C66H125NO10	1092.93763	Positive	M+H	1	9.38
CAR 10:0	Target Compound	C17H33NO4	316.24824	Positive	M+H	1	2
CAR 10:1	Target Compound	C17H31NO4	314.23258	Positive	M+H	1	1.89
CAR 12:0	Target Compound	C19H37NO4	344.27954	Positive	M+H	1	2.21
CAR 12:1	Target Compound	C19H35NO4	342.26389	Positive	M+H	1	2.13
CAR 13:1	Target Compound	C20H37NO4	356.27954	Positive	M+H	1	2.2

CAR 14:0	Target Compound	C21H41NO4	372.31084	Positive	M+H	1	2.37
CAR 14:1	Target Compound	C21H39NO4	370.29519	Positive	M+H	1	2.28
CAR 16:0	Target Compound	C23H45NO4	400.34214	Positive	M+H	1	2.5
CAR 16:1	Target Compound	C23H43NO4	398.32649	Positive	M+H	1	2.42
CAR 18:0	Target Compound	C25H49NO4	428.37344	Positive	M+H	1	2.65
CAR 18:1	Target Compound	C25H47NO4	426.35779	Positive	M+H	1	2.55
CAR 18:2	Target Compound	C25H45NO4	424.34214	Positive	M+H	1	2.45
CAR 20:0	Target Compound	C27H53NO4	456.40474	Positive	M+H	1	2.81
CAR 20:1	Target Compound	C27H51NO4	454.38909	Positive	M+H	1	2.68
CAR 20:2	Target Compound	C27H49NO4	452.37344	Positive	M+H	1	2.56
CAR 20:4	Target Compound	C27H45NO4	448.34214	Positive	M+H	1	2.45
CAR 22:1	Target Compound	C29H55NO4	482.42039	Positive	M+H	1	2.85
CAR 22:2	Target Compound	C29H53NO4	480.40474	Positive	M+H	1	2.71
CAR 8:0	Target Compound	C15H29NO4	288.21693	Positive	M+H	1	1.7
CE 18:1-d7	Internal Standard	D7C45H71O2	675.67794	Positive	M+NH4	1	9.42
Cer 18:0;O2/16:0	Target Compound	C34H69NO3	540.53502	Positive	M+H	1	5.13
Cer 18:0;O2/22:0	Target Compound	C40H81NO3	624.62892	Positive	M+H	1	6.87
Cer 18:0;O2/24:0	Target Compound	C42H85NO3	652.66022	Positive	M+H	1	7.48
Cer 18:0;O2/24:1	Target Compound	C42H83NO3	650.64457	Positive	M+H	1	6.89
Cer 18:1;O2/16:0	Target Compound	C34H67NO3	538.51937	Positive	M+H	1	4.92
Cer 18:1;O2/16:0-d7	Internal Standard	D7C34H60NO3	545.56331	Positive	M+H	1	4.92
Cer 18:1;O2/18:0	Target Compound	C36H71NO3	566.55067	Positive	M+H	1	5.48
Cer 18:1;O2/20:0	Target Compound	C38H75NO3	594.58197	Positive	M+H	1	6.16
Cer 18:1;O2/21:0	Target Compound	C39H77NO3	608.59762	Positive	M+H	1	6.45
Cer 18:1;O2/22:0	Target Compound	C40H79NO3	622.61327	Positive	M+H	1	6.67
Cer 18:1;O2/23:0	Target Compound	C41H81NO3	636.62892	Positive	M+H	1	6.98
Cer 18:1;O2/24:0	Target Compound	C42H83NO3	650.64457	Positive	M+H	1	7.27
Cer 18:1;O2/24:1	Target Compound	C42H81NO3	648.62892	Positive	M+H	1	6.69
Cer 18:1;O2/26:0	Target Compound	C44H87NO3	678.67587	Positive	M+H	1	7.82
Cer 18:1;O2/36:10	Target Compound	C54H87NO3	798.67587	Positive	M+H	1	6.32
Cer 18:1;O2/38:10	Target Compound	C56H91NO3	826.70717	Positive	M+H	1	6.92
Cer 18:2;O2/16:0	Target Compound	C34H65NO3	536.50372	Positive	M+H	1	4.37
Cer 18:2;O2/22:0	Target Compound	C40H77NO3	620.59762	Positive	M+H	1	6.2
Cer 18:2;O2/23:0	Target Compound	C41H79NO3	634.61327	Positive	M+H	1	6.51
Cer 18:2;O2/24:1	Target Compound	C42H79NO3	646.61327	Positive	M+H	1	6.21
Cholesterol-d7	Internal Standard	D7C27H39O	376.39552	Positive	M-OH-	1	3.93
DG 15:0/18:1-d7	Internal Standard	D7C36H61O5	605.58444	Positive	M+NH4	1	5.59
DG 30:1	Target Compound	C33H62O5	556.49355	Positive	M+NH4	1	4.8
DG 31:0	Target Compound	C34H66O5	572.52485	Positive	M+NH4	1	5.5
DG 31:1	Target Compound	C34H64O5	570.5092	Positive	M+NH4	1	5.05
DG 32:0	Target Compound	C35H68O5	586.5405	Positive	M+NH4	1	5.8
DG 32:1	Target Compound	C35H66O5	584.52485	Positive	M+NH4	1	5.35
DG 32:2	Target Compound	C35H64O5	582.5092	Positive	M+NH4	1	4.94
DG 33:0	Target Compound	C36H70O5	600.55615	Positive	M+NH4	1	6.05
DG 33:1	Target Compound	C36H68O5	598.5405	Positive	M+NH4	1	5.6
DG 34:0	Target Compound	C37H72O5	614.5718	Positive	M+NH4	1	6.35
DG 34:1	Target Compound	C37H70O5	612.55615	Positive	M+NH4	1	5.9
DG 34:2	Target Compound	C37H68O5	610.5405	Positive	M+NH4	1	5.48
DG 34:3	Target Compound	C37H66O5	608.52485	Positive	M+NH4	1	5.05
DG 35:1	Target Compound	C38H72O5	626.5718	Positive	M+NH4	1	6.2
DG 35:2	Target Compound	C38H70O5	624.55615	Positive	M+NH4	1	5.76
DG 36:0	Target Compound	C39H76O5	642.6031	Positive	M+NH4	1	6.95
DG 36:1	Target Compound	C39H74O5	640.58745	Positive	M+NH4	1	6.5
DG 36:2	Target Compound	C39H72O5	638.5718	Positive	M+NH4	1	6.05
DG 36:3	Target Compound	C39H70O5	636.55615	Positive	M+NH4	1	5.6
DG 36:4	Target Compound	C39H68O5	634.5405	Positive	M+NH4	1	5.09
DG 36:5	Target Compound	C39H66O5	632.52485	Positive	M+NH4	1	4.7
DG 38:1	Target Compound	C41H78O5	668.61875	Positive	M+NH4	1	6.8
DG 38:2	Target Compound	C41H76O5	666.6031	Positive	M+NH4	1	6.33
DG 38:3	Target Compound	C41H74O5	664.58745	Positive	M+NH4	1	5.9
DG 38:4	Target Compound	C41H72O5	662.5718	Positive	M+NH4	1	5.9
DG 38:5	Target Compound	C41H70O5	660.55615	Positive	M+NH4	1	5.38
DG 38:6	Target Compound	C41H68O5	658.5405	Positive	M+NH4	1	4.97
DG 40:6	Target Compound	C43H72O5	686.5718	Positive	M+NH4	1	5.4
DG 40:7	Target Compound	C43H70O5	684.55615	Positive	M+NH4	1	5.19
DG 40:8	Target Compound	C43H68O5	682.5405	Positive	M+NH4	1	4.76
DG 44:8	Target Compound	C47H76O5	738.6031	Positive	M+NH4	1	5.85
Hex2Cer 18:1;O2/16:0	Target Compound	C46H87NO13	862.62502	Positive	M+H	1	4.24
Hex2Cer 18:1;O2/24:0	Target Compound	C54H103NO13	974.75022	Positive	M+H	1	6.37

Hex2Cer 18:1;O2/24:1	Target Compound	C54H101NO13	972.73457	Positive	M+H	1	5.76
HexCer 18:0;O2/18:0	Target Compound	C42H83NO8	730.61914	Positive	M+H	1	5.15
HexCer 18:0;O2/22:0	Target Compound	C46H91NO8	786.68175	Positive	M+H	1	6.28
HexCer 18:0;O2/22:0;O	Target Compound	C46H91NO9	802.67666	Positive	M+H	1	6.22
HexCer 18:0;O2/23:0	Target Compound	C47H93NO8	800.6974	Positive	M+H	1	6.58
HexCer 18:0;O2/24:0	Target Compound	C48H95NO8	814.71305	Positive	M+H	1	6.87
HexCer 18:0;O2/24:0;O	Target Compound	C48H95NO9	830.70796	Positive	M+H	1	6.84
HexCer 18:1;O2/18:0	Target Compound	C42H81NO8	728.60349	Positive	M+H	1	4.94
HexCer 18:1;O2/18:0;O	Target Compound	C42H81NO9	744.59841	Positive	M+H	1	4.88
HexCer 18:1;O2/20:0	Target Compound	C44H85NO8	756.63479	Positive	M+H	1	5.52
HexCer 18:1;O2/20:0;O	Target Compound	C44H85NO9	772.62971	Positive	M+H	1	5.42
HexCer 18:1;O2/21:0;O	Target Compound	C45H87NO9	786.64536	Positive	M+H	1	5.7
HexCer 18:1;O2/22:0	Target Compound	C46H89NO8	784.6661	Positive	M+H	1	6.07
HexCer 18:1;O2/22:0;O	Target Compound	C46H89NO9	800.66101	Positive	M+H	1	5.95
HexCer 18:1;O2/22:1	Target Compound	C46H87NO8	782.65045	Positive	M+H	1	5.51
HexCer 18:1;O2/22:1;O	Target Compound	C46H87NO9	798.64536	Positive	M+H	1	5.46
HexCer 18:1;O2/23:0;O	Target Compound	C47H91NO9	814.67666	Positive	M+H	1	6.3
HexCer 18:1;O2/23:1	Target Compound	C47H89NO8	796.6661	Positive	M+H	1	5.79
HexCer 18:1;O2/23:1;O	Target Compound	C47H89NO9	812.66101	Positive	M+H	1	5.8
HexCer 18:1;O2/24:0	Target Compound	C48H93NO8	812.6974	Positive	M+H	1	6.71
HexCer 18:1;O2/24:0;O	Target Compound	C48H93NO9	828.69231	Positive	M+H	1	6.6
HexCer 18:1;O2/24:1	Target Compound	C48H91NO8	810.68175	Positive	M+H	1	6.06
HexCer 18:1;O2/24:1;O	Target Compound	C48H91NO9	826.67666	Positive	M+H	1	6
HexCer 18:1;O2/24:2	Target Compound	C48H89NO8	808.6661	Positive	M+H	1	5.6
HexCer 18:1;O2/24:2;O	Target Compound	C48H89NO9	824.66101	Positive	M+H	1	5.52
HexCer 18:1;O2/25:0;O	Target Compound	C49H95NO9	842.70796	Positive	M+H	1	6.84
HexCer 18:1;O2/25:1	Target Compound	C49H93NO8	824.6974	Positive	M+H	1	6.29
HexCer 18:1;O2/25:1;O	Target Compound	C49H93NO9	840.69231	Positive	M+H	1	6.28
HexCer 18:1;O2/40:2;O	Target Compound	C64H121NO9	1048.91141	Positive	M+H	1	9.07
HexCer 18:1;O2/42:2;O	Target Compound	C66H125NO9	1076.94271	Positive	M+H	1	9.44
HexCer 18:2;O2/24:0	Target Compound	C48H91NO8	810.68175	Positive	M+H	1	6.16
LacCer 18:1;O2/17:0	Internal Standard	C47H89NO13	876.64067	Positive	M+H	1	4.46
LPC 14:0	Target Compound	C22H46NO7P	468.30847	Positive	M+H	1	2.45
LPC 15:0	Target Compound	C23H48NO7P	482.32412	Positive	M+H	1	2.53
LPC 16:0	Target Compound	C24H50NO7P	496.33977	Positive	M+H	1	2.61
LPC 16:1	Target Compound	C24H48NO7P	494.32412	Positive	M+H	1	2.49
LPC 17:0	Target Compound	C25H52NO7P	510.35542	Positive	M+H	1	2.68
LPC 18:0	Target Compound	C26H54NO7P	524.37107	Positive	M+H	1	2.78
LPC 18:1	Target Compound	C26H52NO7P	522.35542	Positive	M+H	1	2.65
LPC 18:1-d7	Internal Standard	D7C26H45NO7P	529.39935	Positive	M+H	1	2.65
LPC 18:2	Target Compound	C26H50NO7P	520.33977	Positive	M+H	1	2.53
LPC 18:3	Target Compound	C26H48NO7P	518.32412	Positive	M+H	1	2.45
LPC 19:0	Target Compound	C27H56NO7P	538.38672	Positive	M+H	1	2.88
LPC 19:1	Target Compound	C27H54NO7P	536.37107	Positive	M+H	1	2.73
LPC 20:0	Target Compound	C28H58NO7P	552.40237	Positive	M+H	1	3
LPC 20:1	Target Compound	C28H56NO7P	550.38672	Positive	M+H	1	2.81
LPC 20:2	Target Compound	C28H54NO7P	548.37107	Positive	M+H	1	2.68
LPC 20:3	Target Compound	C28H52NO7P	546.35542	Positive	M+H	1	2.78
LPC 20:4	Target Compound	C28H50NO7P	544.33977	Positive	M+H	1	2.52
LPC 20:5	Target Compound	C28H48NO7P	542.32412	Positive	M+H	1	2.4
LPC 22:0	Target Compound	C30H62NO7P	580.43367	Positive	M+H	1	3.3
LPC 22:4	Target Compound	C30H54NO7P	572.37107	Positive	M+H	1	2.64
LPC 22:5	Target Compound	C30H52NO7P	570.35542	Positive	M+H	1	2.53
LPC 22:6	Target Compound	C30H50NO7P	568.33977	Positive	M+H	1	2.49
LPC 24:0	Target Compound	C32H66NO7P	608.46497	Positive	M+H	1	3.65
LPC 24:1	Target Compound	C32H64NO7P	606.44932	Positive	M+H	1	3.3
LPC O-16:0-d4	Internal Standard	D4C24H48NO6P	486.38561	Positive	M+H	1	2.69
LPC O-24:1	Target Compound	C32H66NO6P	592.47005	Positive	M+H	1	3.48
LPE 18:1-d7	Internal Standard	D7C23H39NO7P	487.3524	Positive	M+H	1	2.67
LPG 17:1	Internal Standard	C23H45O9P	514.31394	Positive	M+NH4	1	2.48
LPI 17:1	Internal Standard	C26H49O12P	602.32999	Positive	M+NH4	1	2.45
PC 15:0/18:1-d7	Internal Standard	D7C41H73NO8P	753.61337	Positive	M+H	1	4.47
PC 25:0	Target Compound	C33H66NO8P	636.45988	Positive	M+H	1	3.2
PC 28:0	Target Compound	C36H72NO8P	678.50683	Positive	M+H	1	3.8
PC 28:1	Target Compound	C36H70NO8P	676.49118	Positive	M+H	1	3.52
PC 29:0	Target Compound	C37H74NO8P	692.52248	Positive	M+H	1	3.98
PC 30:0	Target Compound	C38H76NO8P	706.53813	Positive	M+H	1	4.17
PC 30:1	Target Compound	C38H74NO8P	704.52248	Positive	M+H	1	3.87
PC 30:2	Target Compound	C38H72NO8P	702.50683	Positive	M+H	1	3.6

PC 31:0	Target Compound	C39H78NO8P	720.55378	Positive	M+H	1	4.39
PC 31:1	Target Compound	C39H76NO8P	718.53813	Positive	M+H	1	4.05
PC 31:2	Target Compound	C39H74NO8P	716.52248	Positive	M+H	1	3.72
PC 32:0	Target Compound	C40H80NO8P	734.56943	Positive	M+H	1	4.59
PC 32:1	Target Compound	C40H78NO8P	732.55378	Positive	M+H	1	4.26
PC 32:2	Target Compound	C40H76NO8P	730.53813	Positive	M+H	1	3.93
PC 33:0	Target Compound	C41H82NO8P	748.58508	Positive	M+H	1	4.8
PC 33:1	Target Compound	C41H80NO8P	746.56943	Positive	M+H	1	4.47
PC 33:2	Target Compound	C41H78NO8P	744.55378	Positive	M+H	1	4.14
PC 33:3	Target Compound	C41H76NO8P	742.53813	Positive	M+H	1	3.77
PC 34:0	Target Compound	C42H84NO8P	762.60073	Positive	M+H	1	5.1
PC 34:0;O	Target Compound	C42H82NO9P	776.58	Positive	M+H	1	4.2
PC 34:1	Target Compound	C42H82NO8P	760.58508	Positive	M+H	1	4.69
PC 34:2	Target Compound	C42H80NO8P	758.56943	Positive	M+H	1	4.33
PC 34:3	Target Compound	C42H78NO8P	756.55378	Positive	M+H	1	4.03
PC 34:4	Target Compound	C42H76NO8P	754.53813	Positive	M+H	1	3.9
PC 34:5	Target Compound	C42H74NO8P	752.52248	Positive	M+H	1	3.62
PC 35:0	Target Compound	C43H86NO8P	776.61638	Positive	M+H	1	5.3
PC 35:1	Target Compound	C43H84NO8P	774.60073	Positive	M+H	1	4.94
PC 35:2	Target Compound	C43H82NO8P	772.58508	Positive	M+H	1	4.56
PC 35:3	Target Compound	C43H80NO8P	770.56943	Positive	M+H	1	4.23
PC 35:4	Target Compound	C43H78NO8P	768.55378	Positive	M+H	1	4.08
PC 36:0	Target Compound	C44H88NO8P	790.63203	Positive	M+H	1	5.62
PC 36:1	Target Compound	C44H86NO8P	788.61638	Positive	M+H	1	5.21
PC 36:2	Target Compound	C44H84NO8P	786.60073	Positive	M+H	1	4.81
PC 36:3	Target Compound	C44H82NO8P	784.58508	Positive	M+H	1	4.46
PC 36:4	Target Compound	C44H80NO8P	782.56943	Positive	M+H	1	4.28
PC 36:4;O	Target Compound	C44H80NO9P	798.56435	Positive	M+H	1	3.84
PC 36:5	Target Compound	C44H78NO8P	780.55378	Positive	M+H	1	3.98
PC 36:6	Target Compound	C44H76NO8P	778.53813	Positive	M+H	1	3.74
PC 37:1	Target Compound	C45H88NO8P	802.63203	Positive	M+H	1	5.48
PC 37:2	Target Compound	C45H86NO8P	800.61638	Positive	M+H	1	5.06
PC 37:3	Target Compound	C45H84NO8P	798.60073	Positive	M+H	1	4.66
PC 37:4	Target Compound	C45H82NO8P	796.58508	Positive	M+H	1	4.51
PC 37:6	Target Compound	C45H78NO8P	792.55378	Positive	M+H	1	3.93
PC 38:0	Target Compound	C46H92NO8P	818.66333	Positive	M+H	1	6.21
PC 38:1	Target Compound	C46H90NO8P	816.64768	Positive	M+H	1	5.68
PC 38:2	Target Compound	C46H88NO8P	814.63203	Positive	M+H	1	5.24
PC 38:3	Target Compound	C46H86NO8P	812.61638	Positive	M+H	1	4.93
PC 38:4	Target Compound	C46H84NO8P	810.60073	Positive	M+H	1	4.75
PC 38:5	Target Compound	C46H82NO8P	808.58508	Positive	M+H	1	4.39
PC 38:6	Target Compound	C46H80NO8P	806.56943	Positive	M+H	1	4.14
PC 38:7	Target Compound	C46H78NO8P	804.55378	Positive	M+H	1	3.85
PC 39:1	Target Compound	C47H92NO8P	830.66333	Positive	M+H	1	6.03
PC 39:2	Target Compound	C47H90NO8P	828.64768	Positive	M+H	1	5.6
PC 39:3	Target Compound	C47H88NO8P	826.63203	Positive	M+H	1	5.19
PC 39:4	Target Compound	C47H86NO8P	824.61638	Positive	M+H	1	5.01
PC 39:5	Target Compound	C47H84NO8P	822.60073	Positive	M+H	1	4.55
PC 39:6	Target Compound	C47H82NO8P	820.58508	Positive	M+H	1	4.31
PC 39:7	Target Compound	C47H80NO8P	818.56943	Positive	M+H	1	4.02
PC 40:0	Target Compound	C48H96NO8P	846.69463	Positive	M+H	1	6.81
PC 40:1	Target Compound	C48H94NO8P	844.67898	Positive	M+H	1	6.34
PC 40:2	Target Compound	C48H92NO8P	842.66333	Positive	M+H	1	5.9
PC 40:4	Target Compound	C48H88NO8P	838.63203	Positive	M+H	1	5.25
PC 40:6	Target Compound	C48H84NO8P	834.60073	Positive	M+H	1	4.56
PC 40:7	Target Compound	C48H82NO8P	832.58508	Positive	M+H	1	4.2
PC 40:8	Target Compound	C48H80NO8P	830.56943	Positive	M+H	1	3.93
PC 41:1	Target Compound	C49H96NO8P	858.69463	Positive	M+H	1	6.6
PC 41:2	Target Compound	C49H94NO8P	856.67898	Positive	M+H	1	6.18
PC 41:6	Target Compound	C49H86NO8P	848.61638	Positive	M+H	1	4.76
PC 42:1	Target Compound	C50H98NO8P	872.71028	Positive	M+H	1	6.94
PC 42:10	Target Compound	C50H80NO8P	854.56943	Positive	M+H	1	3.81
PC 42:2	Target Compound	C50H96NO8P	870.69463	Positive	M+H	1	6.47
PC 42:4	Target Compound	C50H92NO8P	866.66333	Positive	M+H	1	5.6
PC 42:5	Target Compound	C50H90NO8P	864.64768	Positive	M+H	1	5.1
PC 42:6	Target Compound	C50H88NO8P	862.63203	Positive	M+H	1	4.86
PC 42:7	Target Compound	C50H86NO8P	860.61638	Positive	M+H	1	4.62
PC 42:8	Target Compound	C50H84NO8P	858.60073	Positive	M+H	1	4.23
PC 42:9	Target Compound	C50H82NO8P	856.58508	Positive	M+H	1	4

PC 43:1	Target Compound	C51H100NO8P	886.72593	Positive	M+H	1	7.19
PC 43:2	Target Compound	C51H98NO8P	884.71028	Positive	M+H	1	6.78
PC 44:1	Target Compound	C52H102NO8P	900.74158	Positive	M+H	1	7.47
PC 44:10	Target Compound	C52H84NO8P	882.60073	Positive	M+H	1	4.23
PC 44:11	Target Compound	C52H82NO8P	880.58508	Positive	M+H	1	3.91
PC 44:12	Target Compound	C52H80NO8P	878.56943	Positive	M+H	1	3.6
PC 44:2	Target Compound	C52H100NO8P	898.72593	Positive	M+H	1	7.05
PC 44:4	Target Compound	C52H96NO8P	894.69463	Positive	M+H	1	6.35
PC 44:5	Target Compound	C52H94NO8P	892.67898	Positive	M+H	1	5.81
PC 44:8	Target Compound	C52H88NO8P	886.63203	Positive	M+H	1	4.58
PC O-16:0_16:0	Target Compound	C40H82NO7P	720.59017	Positive	M+H	1	4.94
PC O-16:0_18:2	Target Compound	C42H82NO7P	744.59017	Positive	M+H	1	5.02
PC O-16:0_20:4	Target Compound	C44H82NO7P	768.59017	Positive	M+H	1	4.59
PC O-16:1_16:0	Target Compound	C40H80NO7P	718.57452	Positive	M+H	1	4.89
PC O-16:1_18:1	Target Compound	C42H82NO7P	744.59017	Positive	M+H	1	4.66
PC O-16:1_18:2	Target Compound	C42H80NO7P	742.57452	Positive	M+H	1	4.6
PC O-16:1_20:4	Target Compound	C44H80NO7P	766.57452	Positive	M+H	1	4.53
PC O-18:0/18:1-d9	Internal Standard	D9C44H77NO7P	781.67796	Positive	M+H	1	5.51
PC O-18:0_20:4	Target Compound	C46H86NO7P	796.62147	Positive	M+H	1	5.1
PC O-18:1_18:1	Target Compound	C44H86NO7P	772.62147	Positive	M+H	1	5.16
PC O-18:1_18:2	Target Compound	C44H84NO7P	770.60582	Positive	M+H	1	4.75
PC O-18:1_20:4	Target Compound	C46H84NO7P	794.60582	Positive	M+H	1	4.65
PC O-30:0	Target Compound	C38H78NO7P	692.55887	Positive	M+H	1	5.84
PC O-30:1	Target Compound	C38H76NO7P	690.54322	Positive	M+H	1	5.34
PC O-31:0	Target Compound	C39H80NO7P	706.57452	Positive	M+H	1	5.26
PC O-31:1	Target Compound	C39H78NO7P	704.55887	Positive	M+H	1	6.47
PC O-32:2	Target Compound	C40H78NO7P	716.55887	Positive	M+H	1	4.6
PC O-33:0	Target Compound	C41H84NO7P	734.60582	Positive	M+H	1	4.35
PC O-33:1	Target Compound	C41H82NO7P	732.59017	Positive	M+H	1	4.59
PC O-34:0	Target Compound	C42H86NO7P	748.62147	Positive	M+H	1	4.86
PC O-34:1	Target Compound	C42H84NO7P	746.60582	Positive	M+H	1	5.05
PC O-34:4	Target Compound	C42H78NO7P	740.55887	Positive	M+H	1	4.34
PC O-36:0	Target Compound	C44H90NO7P	776.65277	Positive	M+H	1	4.9
PC O-36:1	Target Compound	C44H88NO7P	774.63712	Positive	M+H	1	5.51
PC O-36:6	Target Compound	C44H78NO7P	764.55887	Positive	M+H	1	4.4
PC O-37:4	Target Compound	C45H84NO7P	782.60582	Positive	M+H	1	4.86
PC O-37:6	Target Compound	C45H80NO7P	778.57452	Positive	M+H	1	4.5
PC O-38:1	Target Compound	C46H92NO7P	802.66842	Positive	M+H	1	4.9
PC O-38:10	Target Compound	C46H74NO7P	784.52757	Positive	M+H	1	4.15
PC O-38:2_1	Target Compound	C46H90NO7P	800.65277	Positive	M+H	1	6.29
PC O-38:2_2	Target Compound	C46H90NO7P	800.65277	Positive	M+H	1	4.6
PC O-38:3	Target Compound	C46H88NO7P	798.63712	Positive	M+H	1	4.9
PC O-38:6	Target Compound	C46H82NO7P	792.59017	Positive	M+H	1	4.55
PC O-38:7	Target Compound	C46H80NO7P	790.57452	Positive	M+H	1	4.45
PC O-38:9	Target Compound	C46H76NO7P	786.54322	Positive	M+H	1	4.25
PC O-39:1	Target Compound	C47H94NO7P	816.68407	Positive	M+H	1	5.05
PC O-39:6	Target Compound	C47H84NO7P	806.60582	Positive	M+H	1	4.6
PC O-39:8	Target Compound	C47H80NO7P	802.57452	Positive	M+H	1	4.55
PC O-40:10	Target Compound	C48H78NO7P	812.55887	Positive	M+H	1	4.25
PC O-40:11	Target Compound	C48H76NO7P	810.54322	Positive	M+H	1	4.15
PC O-40:2	Target Compound	C48H94NO7P	828.68407	Positive	M+H	1	5.05
PC O-40:4	Target Compound	C48H90NO7P	824.65277	Positive	M+H	1	5.36
PC O-40:5	Target Compound	C48H88NO7P	822.63712	Positive	M+H	1	4.77
PC O-40:6	Target Compound	C48H86NO7P	820.6215	Positive	M+H	1	4.65
PC O-40:7	Target Compound	C48H84NO7P	818.60582	Positive	M+H	1	4.55
PC O-40:8	Target Compound	C48H82NO7P	816.59017	Positive	M+H	1	4.45
PC O-40:9	Target Compound	C48H80NO7P	814.57452	Positive	M+H	1	4.35
PC O-42:10	Target Compound	C50H82NO7P	840.59017	Positive	M+H	1	4.5
PC O-42:3	Target Compound	C50H96NO7P	854.69972	Positive	M+H	1	5.2
PC O-42:4	Target Compound	C50H94NO7P	852.68407	Positive	M+H	1	5.62
PC O-42:5	Target Compound	C50H92NO7P	850.66842	Positive	M+H	1	4.89
PC O-42:6	Target Compound	C50H90NO7P	848.65277	Positive	M+H	1	4.75
PC O-42:7	Target Compound	C50H88NO7P	846.63712	Positive	M+H	1	4.65
PC O-44:5	Target Compound	C52H96NO7P	878.69972	Positive	M+H	1	5.01
PC O-44:6	Target Compound	C52H94NO7P	876.68407	Positive	M+H	1	4.8
PC O-44:7	Target Compound	C52H92NO7P	874.66842	Positive	M+H	1	4.8
PC O-46:7	Target Compound	C54H96NO7P	902.69972	Positive	M+H	1	4.9
PE 15:0/18:1-d7	Internal Standard	D7C38H67NO8P	711.56642	Positive	M+H	1	4.62
PG 15:0/18:1-d7	Internal Standard	D7C39H68O10P	759.58755	Positive	M+NH4	1	4.03

PI 15:0/18:1-d7	Internal Standard	D7C42H72O13P	847.60359	Positive	M+NH4	1	3.91
PS 15:0/18:1-d7	Internal Standard	D7C39H67NO10P	755.55625	Positive	M+H	1	4.07
SE 27:1/14:0	Target Compound	C41H72O2	614.58706	Positive	M+NH4	1	9.05
SE 27:1/15:0	Target Compound	C42H74O2	628.60271	Positive	M+NH4	1	9.25
SE 27:1/16:2	Target Compound	C43H72O2	638.58706	Positive	M+NH4	1	8.62
SE 27:1/17:0	Target Compound	C44H78O2	656.63401	Positive	M+NH4	1	9.65
SE 27:1/17:1	Target Compound	C44H76O2	654.61836	Positive	M+NH4	1	9.3
SE 27:1/18:1	Target Compound	C45H78O2	668.63401	Positive	M+NH4	1	9.44
SE 27:1/18:2	Target Compound	C45H76O2	666.61836	Positive	M+NH4	1	9.04
SE 27:1/18:3	Target Compound	C45H74O2	664.60271	Positive	M+NH4	1	8.61
SE 27:1/20:1	Target Compound	C47H82O2	696.66531	Positive	M+NH4	1	9.8
SE 27:1/20:2	Target Compound	C47H80O2	694.64966	Positive	M+NH4	1	9.46
SE 27:1/20:3	Target Compound	C47H78O2	692.63401	Positive	M+NH4	1	9.08
SE 27:1/20:4	Target Compound	C47H76O2	690.61836	Positive	M+NH4	1	8.76
SE 27:1/20:5	Target Compound	C47H74O2	688.60271	Positive	M+NH4	1	8.34
SE 27:1/22:4	Target Compound	C49H80O2	718.64966	Positive	M+NH4	1	9.14
SE 27:1/22:5	Target Compound	C49H78O2	716.63401	Positive	M+NH4	1	8.77
SE 27:1/22:6	Target Compound	C49H76O2	714.61836	Positive	M+NH4	1	8.46
SE 27:1/24:6	Target Compound	C51H80O2	742.64966	Positive	M+NH4	1	8.9
SM 18:1;O2/18:1-d9	Internal Standard	D9C41H72N2O6P	738.64699	Positive	M+H	1	4.36
SM 30:0;O2	Target Compound	C35H73N2O6P	649.5279	Positive	M+H	1	3.67
SM 30:1;O2	Target Compound	C35H71N2O6P	647.51225	Positive	M+H	1	3.52
SM 32:0;O2	Target Compound	C37H77N2O6P	677.5592	Positive	M+H	1	4.03
SM 32:1;O2	Target Compound	C37H75N2O6P	675.54355	Positive	M+H	1	3.86
SM 32:2;O2	Target Compound	C37H73N2O6P	673.5279	Positive	M+H	1	3.57
SM 33:1;O2	Target Compound	C38H77N2O6P	689.5592	Positive	M+H	1	4.06
SM 33:2;O2	Target Compound	C38H75N2O6P	687.54355	Positive	M+H	1	3.73
SM 34:0;O2	Target Compound	C39H81N2O6P	705.5905	Positive	M+H	1	4.45
SM 34:1;O2	Target Compound	C39H79N2O6P	703.57485	Positive	M+H	1	4.25
SM 34:1;O3	Target Compound	C39H79N2O7P	719.56977	Positive	M+H	1	4.08
SM 34:2;O2	Target Compound	C39H77N2O6P	701.5592	Positive	M+H	1	3.93
SM 35:0;O2	Target Compound	C40H83N2O6P	719.60615	Positive	M+H	1	4.66
SM 35:1;O2	Target Compound	C40H81N2O6P	717.5905	Positive	M+H	1	4.48
SM 35:2;O2	Target Compound	C40H79N2O6P	715.57485	Positive	M+H	1	4.11
SM 36:0;O2	Target Compound	C41H85N2O6P	733.6218	Positive	M+H	1	4.92
SM 36:1;O2	Target Compound	C41H83N2O6P	731.60615	Positive	M+H	1	4.73
SM 36:2;O2	Target Compound	C41H81N2O6P	729.5905	Positive	M+H	1	4.36
SM 36:3;O2	Target Compound	C41H79N2O6P	727.57485	Positive	M+H	1	4.03
SM 37:1;O2	Target Compound	C42H85N2O6P	745.6218	Positive	M+H	1	5.03
SM 37:2;O2	Target Compound	C42H83N2O6P	743.60615	Positive	M+H	1	4.59
SM 38:0;O2	Target Compound	C43H89N2O6P	761.6531	Positive	M+H	1	5.51
SM 38:1;O2	Target Compound	C43H87N2O6P	759.63745	Positive	M+H	1	5.32
SM 38:2;O2	Target Compound	C43H85N2O6P	757.6218	Positive	M+H	1	4.86
SM 38:3;O2	Target Compound	C43H83N2O6P	755.60615	Positive	M+H	1	4.38
SM 39:1;O2	Target Compound	C44H89N2O6P	773.6531	Positive	M+H	1	5.6
SM 39:2;O2	Target Compound	C44H87N2O6P	771.63745	Positive	M+H	1	5.12
SM 40:0;O2	Target Compound	C45H93N2O6P	789.6844	Positive	M+H	1	6.03
SM 40:1;O2	Target Compound	C45H91N2O6P	787.66875	Positive	M+H	1	5.82
SM 40:2;O2	Target Compound	C45H89N2O6P	785.6531	Positive	M+H	1	5.37
SM 40:3;O2	Target Compound	C45H87N2O6P	783.63745	Positive	M+H	1	4.86
SM 40:4;O2	Target Compound	C45H85N2O6P	781.6218	Positive	M+H	1	4.48
SM 41:0;O2	Target Compound	C46H95N2O6P	803.70005	Positive	M+H	1	6.34
SM 41:1;O2	Target Compound	C46H93N2O6P	801.6844	Positive	M+H	1	6.12
SM 41:2;O2	Target Compound	C46H91N2O6P	799.66875	Positive	M+H	1	5.67
SM 41:3;O2	Target Compound	C46H89N2O6P	797.6531	Positive	M+H	1	5.12
SM 42:0;O2	Target Compound	C47H97N2O6P	817.7157	Positive	M+H	1	6.65
SM 42:0;O3	Target Compound	C47H97N2O7P	833.71062	Positive	M+H	1	6.16
SM 42:1;O2	Target Compound	C47H95N2O6P	815.70005	Positive	M+H	1	6.43
SM 42:1;O3	Target Compound	C47H95N2O7P	831.69497	Positive	M+H	1	5.57
SM 42:2;O2	Target Compound	C47H93N2O6P	813.6844	Positive	M+H	1	5.84
SM 42:3;O2	Target Compound	C47H91N2O6P	811.66875	Positive	M+H	1	5.37
SM 42:4;O2	Target Compound	C47H89N2O6P	809.6531	Positive	M+H	1	4.92
SM 42:5;O2	Target Compound	C47H87N2O6P	807.63745	Positive	M+H	1	4.62
SM 43:1;O2	Target Compound	C48H97N2O6P	829.7157	Positive	M+H	1	6.64
SM 43:2;O2	Target Compound	C48H95N2O6P	827.70005	Positive	M+H	1	6.05
SM 43:3;O2	Target Compound	C48H93N2O6P	825.6844	Positive	M+H	1	5.65
SM 44:1;O2	Target Compound	C49H99N2O6P	843.73135	Positive	M+H	1	7.03
SM 44:2;O2	Target Compound	C49H97N2O6P	841.7157	Positive	M+H	1	6.38
SM 44:3;O2	Target Compound	C49H95N2O6P	839.70005	Positive	M+H	1	5.92

ST 27:1;O	Target Compound	C27H46O	369.35158	Positive	M-OH	1	3.96
TG 14:0/16:1/14:0-d5	Internal Standard	C47H83D5O6	771.7233	Positive	M+NH4	1	8.35
TG 15:0/18:1-d7/15:0	Internal Standard	D7C51H89O6	829.79845	Positive	M+NH4	1	9.19
TG 20:0/20:1/20:0-d5	Internal Standard	C63H115D5O6	995.9737	Positive	M+NH4	1	11
TG 24:0	Target Compound	C27H50O6	488.39456	Positive	M+NH4	1	3.7
TG 26:0	Target Compound	C29H54O6	516.42586	Positive	M+NH4	1	4.1
TG 28:0	Target Compound	C31H58O6	544.45717	Positive	M+NH4	1	4.57
TG 34:0	Target Compound	C37H70O6	628.55107	Positive	M+NH4	1	6.23
TG 36:1	Target Compound	C39H72O6	654.56672	Positive	M+NH4	1	6.33
TG 42:0	Target Compound	C45H86O6	740.67627	Positive	M+NH4	1	8.34
TG 42:1	Target Compound	C45H84O6	738.66062	Positive	M+NH4	1	7.93
TG 42:2	Target Compound	C45H82O6	736.64497	Positive	M+NH4	1	7.49
TG 43:0	Target Compound	C46H88O6	754.69192	Positive	M+NH4	1	8.54
TG 44:0	Target Compound	C47H90O6	768.70757	Positive	M+NH4	1	8.77
TG 44:1	Target Compound	C47H88O6	766.69192	Positive	M+NH4	1	8.4
TG 44:2	Target Compound	C47H86O6	764.67627	Positive	M+NH4	1	7.99
TG 44:3	Target Compound	C47H84O6	762.66062	Positive	M+NH4	1	7.57
TG 45:0	Target Compound	C48H92O6	782.72322	Positive	M+NH4	1	8.95
TG 45:1	Target Compound	C48H90O6	780.70757	Positive	M+NH4	1	8.62
TG 46:0	Target Compound	C49H94O6	796.73887	Positive	M+NH4	1	9.16
TG 46:1	Target Compound	C49H92O6	794.72322	Positive	M+NH4	1	8.82
TG 46:2	Target Compound	C49H90O6	792.70757	Positive	M+NH4	1	8.44
TG 46:3	Target Compound	C49H88O6	790.69192	Positive	M+NH4	1	8.04
TG 47:0	Target Compound	C50H96O6	810.75452	Positive	M+NH4	1	9.35
TG 47:1	Target Compound	C50H94O6	808.73887	Positive	M+NH4	1	9.01
TG 47:2	Target Compound	C50H92O6	806.72322	Positive	M+NH4	1	8.66
TG 48:0	Target Compound	C51H98O6	824.77017	Positive	M+NH4	1	9.53
TG 48:1	Target Compound	C51H96O6	822.75452	Positive	M+NH4	1	9.2
TG 48:2	Target Compound	C51H94O6	820.73887	Positive	M+NH4	1	8.86
TG 48:3	Target Compound	C51H92O6	818.72322	Positive	M+NH4	1	8.48
TG 48:4	Target Compound	C51H90O6	816.70757	Positive	M+NH4	1	8.09
TG 49:0	Target Compound	C52H100O6	838.78582	Positive	M+NH4	1	9.7
TG 49:1	Target Compound	C52H98O6	836.77017	Positive	M+NH4	1	9.39
TG 49:2	Target Compound	C52H96O6	834.75452	Positive	M+NH4	1	9.05
TG 49:3	Target Compound	C52H94O6	832.73887	Positive	M+NH4	1	8.7
TG 49:4	Target Compound	C52H92O6	830.72322	Positive	M+NH4	1	8.43
TG 50:0	Target Compound	C53H102O6	852.80147	Positive	M+NH4	1	9.88
TG 50:1	Target Compound	C53H100O6	850.78582	Positive	M+NH4	1	9.57
TG 50:2	Target Compound	C53H98O6	848.77017	Positive	M+NH4	1	9.25
TG 50:3	Target Compound	C53H96O6	846.75452	Positive	M+NH4	1	8.9
TG 50:4	Target Compound	C53H94O6	844.73887	Positive	M+NH4	1	8.54
TG 50:5	Target Compound	C53H92O6	842.72322	Positive	M+NH4	1	8.15
TG 51:0	Target Compound	C54H104O6	866.81712	Positive	M+NH4	1	10.02
TG 51:1	Target Compound	C54H102O6	864.80147	Positive	M+NH4	1	9.74
TG 51:2	Target Compound	C54H100O6	862.78582	Positive	M+NH4	1	9.45
TG 51:3	Target Compound	C54H98O6	860.77017	Positive	M+NH4	1	9.11
TG 51:4	Target Compound	C54H96O6	858.75452	Positive	M+NH4	1	8.75
TG 52:0	Target Compound	C55H106O6	880.83277	Positive	M+NH4	1	10.17
TG 52:1	Target Compound	C55H104O6	878.81712	Positive	M+NH4	1	9.91
TG 52:2	Target Compound	C55H102O6	876.80147	Positive	M+NH4	1	9.61
TG 52:3	Target Compound	C55H100O6	874.78582	Positive	M+NH4	1	9.29
TG 52:4	Target Compound	C55H98O6	872.77017	Positive	M+NH4	1	8.95
TG 52:5	Target Compound	C55H96O6	870.75452	Positive	M+NH4	1	8.6
TG 52:6	Target Compound	C55H94O6	868.73887	Positive	M+NH4	1	8.35
TG 52:7	Target Compound	C55H92O6	866.72322	Positive	M+NH4	1	8.05
TG 53:0	Target Compound	C56H108O6	894.84842	Positive	M+NH4	1	10.33
TG 53:1	Target Compound	C56H106O6	892.83277	Positive	M+NH4	1	10.06
TG 53:2	Target Compound	C56H104O6	890.81712	Positive	M+NH4	1	9.77
TG 53:3	Target Compound	C56H102O6	888.80147	Positive	M+NH4	1	9.47
TG 53:4	Target Compound	C56H100O6	886.78582	Positive	M+NH4	1	9.1
TG 53:5	Target Compound	C56H98O6	884.77017	Positive	M+NH4	1	8.8
TG 54:0	Target Compound	C57H110O6	908.86407	Positive	M+NH4	1	10.44
TG 54:1	Target Compound	C57H108O6	906.84842	Positive	M+NH4	1	10.23
TG 54:2	Target Compound	C57H106O6	904.83277	Positive	M+NH4	1	9.95
TG 54:3	Target Compound	C57H104O6	902.81712	Positive	M+NH4	1	9.67
TG 54:4	Target Compound	C57H102O6	900.80147	Positive	M+NH4	1	9.33
TG 54:5	Target Compound	C57H100O6	898.78582	Positive	M+NH4	1	9.05
TG 54:6	Target Compound	C57H98O6	896.77017	Positive	M+NH4	1	8.76
TG 54:7	Target Compound	C57H96O6	894.75452	Positive	M+NH4	1	8.46

TG 55:0	Target Compound	C58H112O6	922.87972	Positive	M+NH4	1	10.66
TG 55:1	Target Compound	C58H110O6	920.86407	Positive	M+NH4	1	10.37
TG 55:2	Target Compound	C58H108O6	918.84842	Positive	M+NH4	1	10.1
TG 55:3	Target Compound	C58H106O6	916.83277	Positive	M+NH4	1	9.82
TG 55:4	Target Compound	C58H104O6	914.81712	Positive	M+NH4	1	9.53
TG 55:5	Target Compound	C58H102O6	912.80147	Positive	M+NH4	1	9.28
TG 55:6	Target Compound	C58H100O6	910.78582	Positive	M+NH4	1	9
TG 56:0	Target Compound	C59H114O6	936.89537	Positive	M+NH4	1	10.79
TG 56:1	Target Compound	C59H112O6	934.87972	Positive	M+NH4	1	10.52
TG 56:2	Target Compound	C59H110O6	932.86407	Positive	M+NH4	1	10.26
TG 56:3	Target Compound	C59H108O6	930.84842	Positive	M+NH4	1	9.98
TG 56:4	Target Compound	C59H106O6	928.83277	Positive	M+NH4	1	9.69
TG 56:5	Target Compound	C59H104O6	926.81712	Positive	M+NH4	1	9.53
TG 56:6	Target Compound	C59H102O6	924.80147	Positive	M+NH4	1	9.2
TG 56:7	Target Compound	C59H100O6	922.78582	Positive	M+NH4	1	8.92
TG 56:8	Target Compound	C59H98O6	920.77017	Positive	M+NH4	1	8.57
TG 56:9	Target Compound	C59H96O6	918.75452	Positive	M+NH4	1	8.21
TG 57:1	Target Compound	C60H114O6	948.89537	Positive	M+NH4	1	10.66
TG 57:2	Target Compound	C60H112O6	946.87972	Positive	M+NH4	1	10.4
TG 57:3	Target Compound	C60H110O6	944.86407	Positive	M+NH4	1	10.13
TG 57:4	Target Compound	C60H108O6	942.84842	Positive	M+NH4	1	9.85
TG 58:1	Target Compound	C61H116O6	962.91102	Positive	M+NH4	1	10.8
TG 58:10	Target Compound	C61H98O6	944.77017	Positive	M+NH4	1	8.4
TG 58:11	Target Compound	C61H96O6	942.75452	Positive	M+NH4	1	8
TG 58:2	Target Compound	C61H114O6	960.89537	Positive	M+NH4	1	10.55
TG 58:3	Target Compound	C61H112O6	958.87972	Positive	M+NH4	1	10.28
TG 58:4	Target Compound	C61H110O6	956.86407	Positive	M+NH4	1	10
TG 58:5	Target Compound	C61H108O6	954.84842	Positive	M+NH4	1	9.74
TG 58:6	Target Compound	C61H106O6	952.83277	Positive	M+NH4	1	9.47
TG 58:7	Target Compound	C61H104O6	950.81712	Positive	M+NH4	1	9.17
TG 58:8	Target Compound	C61H102O6	948.80147	Positive	M+NH4	1	9
TG 58:9	Target Compound	C61H100O6	946.78582	Positive	M+NH4	1	8.66
TG 59:2	Target Compound	C62H116O6	974.91102	Positive	M+NH4	1	10.69
TG 59:3	Target Compound	C62H114O6	972.89537	Positive	M+NH4	1	10.42
TG 59:4	Target Compound	C62H112O6	970.87972	Positive	M+NH4	1	10.15
TG 60:10	Target Compound	C63H102O6	972.80147	Positive	M+NH4	1	8.85
TG 60:11	Target Compound	C63H100O6	970.78582	Positive	M+NH4	1	8.53
TG 60:12	Target Compound	C63H98O6	968.77017	Positive	M+NH4	1	8.2
TG 60:2	Target Compound	C63H118O6	988.92667	Positive	M+NH4	1	10.82
TG 60:3	Target Compound	C63H116O6	986.91102	Positive	M+NH4	1	10.55
TG 60:4	Target Compound	C63H114O6	984.89537	Positive	M+NH4	1	10.29
TG 60:5	Target Compound	C63H112O6	982.87972	Positive	M+NH4	1	10.02
TG 60:6	Target Compound	C63H110O6	980.86407	Positive	M+NH4	1	9.75
TG 60:7	Target Compound	C63H108O6	978.84842	Positive	M+NH4	1	9.59
TG 60:8	Target Compound	C63H106O6	976.83277	Positive	M+NH4	1	9.28
TG 62:2	Target Compound	C65H122O6	1016.95797	Positive	M+NH4	1	11.07
TG 63:3	Target Compound	C65H120O6	1014.94232	Positive	M+NH4	1	10.85
TG 70:3	Target Compound	C73H136O6	1127.06752	Positive	M+NH4	1	11.71
TG 72:3	Target Compound	C75H140O6	1155.09882	Positive	M+NH4	1	11.93
TG O-50:1	Target Compound	C53H102O5	836.80655	Positive	M+NH4	1	10.04
TG O-50:2	Target Compound	C55H104O5	862.8222	Positive	M+NH4	1	10.06
TG O-50:3	Target Compound	C55H102O5	860.80655	Positive	M+NH4	1	9.78
TG O-52:3	Target Compound	C57H106O5	888.83785	Positive	M+NH4	1	10.09
Tributyl citrate	Target Compound	C18H32O7	361.22208	Positive	M+H	1	2.47
Ubiquinone-10	Target Compound	C59H90O4	863.69119	Positive	M+H	1	8.34
Arachidonic acid-d8	Internal Standard	D8C20H24O2	311.28317	Negative	M-H	1	2.89
Cer 18:1;O2/16:0-d7	Internal Standard	D7C34H60NO3	589.55423	Negative	M+HCO2	1	4.9
FA 14:0	Target Compound	C14H28O2	227.20165	Negative	M-H	1	2.28
FA 15:0	Target Compound	C15H30O2	241.2173	Negative	M-H	1	2.49
FA 16:0	Target Compound	C16H32O2	255.23295	Negative	M-H	1	3.08
FA 16:1	Target Compound	C16H30O2	253.2173	Negative	M-H	1	2.88
FA 17:0	Target Compound	C17H34O2	269.2486	Negative	M-H	1	3.23
FA 17:1	Target Compound	C17H32O2	267.23295	Negative	M-H	1	3.03
FA 18:0	Target Compound	C18H36O2	283.26425	Negative	M-H	1	3.41
FA 18:1	Target Compound	C18H34O2	281.2486	Negative	M-H	1	3.14
FA 18:2	Target Compound	C18H32O2	279.23295	Negative	M-H	1	2.94
FA 18:3	Target Compound	C18H30O2	277.2173	Negative	M-H	1	2.78
FA 18:4	Target Compound	C18H28O2	275.20165	Negative	M-H	1	2.64
FA 20:1	Target Compound	C20H38O2	309.2799	Negative	M-H	1	3.46

FA 20:2	Target Compound	C20H36O2	307.26425	Negative	M-H	1	3.21
FA 20:4	Target Compound	C20H32O2	303.23295	Negative	M-H	1	2.89
FA 20:5	Target Compound	C20H30O2	301.2173	Negative	M-H	1	2.75
FA 22:4	Target Compound	C22H36O2	331.26425	Negative	M-H	1	3.09
FA 22:6	Target Compound	C22H32O2	327.23295	Negative	M-H	1	2.83
FA 24:0	Target Compound	C24H48O2	367.35815	Negative	M-H	1	5.29
FA 24:1	Target Compound	C24H46O2	365.3425	Negative	M-H	1	4.4
LPC 18:1-d7	Internal Standard	D7C26H45NO7P	573.39028	Negative	M+HCO2	1	2.65
LPC O-16:0	Target Compound	C24H52NO6P	526.35143	Negative	M+HCO2	1	2.69
LPC O-16:0-d4	Internal Standard	D4C24H48NO6P	530.37653	Negative	M+HCO2	1	2.69
LPC O-16:1	Target Compound	C24H50NO6P	524.33578	Negative	M+HCO2	1	2.69
LPC O-17:1	Target Compound	C25H52NO6P	538.35143	Negative	M+HCO2	1	2.76
LPC O-18:0	Target Compound	C26H56NO6P	554.38273	Negative	M+HCO2	1	2.89
LPC O-18:1	Target Compound	C26H54NO6P	552.36708	Negative	M+HCO2	1	2.76
LPC O-18:2	Target Compound	C26H52NO6P	550.35143	Negative	M+HCO2	1	2.72
LPC O-20:0	Target Compound	C28H58NO6P	580.39838	Negative	M+HCO2	1	2.93
LPE 16:0	Target Compound	C21H44NO7P	452.27826	Negative	M-H	1	2.64
LPE 16:1	Target Compound	C21H42NO7P	450.26261	Negative	M-H	1	2.53
LPE 17:0	Target Compound	C22H46NO7P	466.29391	Negative	M-H	1	2.73
LPE 17:2	Target Compound	C22H42NO7P	462.26261	Negative	M-H	1	2.5
LPE 18:0	Target Compound	C23H48NO7P	480.30956	Negative	M-H	1	2.82
LPE 18:1	Target Compound	C23H46NO7P	478.29391	Negative	M-H	1	2.69
LPE 18:1-d7	Internal Standard	D7C23H39NO7P	485.33785	Negative	M-H	1	2.68
LPE 18:2	Target Compound	C23H44NO7P	476.27826	Negative	M-H	1	2.57
LPE 18:3	Target Compound	C23H42NO7P	474.26261	Negative	M-H	1	2.47
LPE 20:0	Target Compound	C25H52NO7P	508.34086	Negative	M-H	1	3.06
LPE 20:1	Target Compound	C25H50NO7P	506.32521	Negative	M-H	1	2.86
LPE 20:2	Target Compound	C25H48NO7P	504.30956	Negative	M-H	1	2.74
LPE 20:3	Target Compound	C25H46NO7P	502.29391	Negative	M-H	1	2.62
LPE 20:4	Target Compound	C25H44NO7P	500.27826	Negative	M-H	1	2.54
LPE 20:5	Target Compound	C25H42NO7P	498.26261	Negative	M-H	1	2.45
LPE 22:0	Target Compound	C27H56NO7P	536.37216	Negative	M-H	1	3.15
LPE 22:3	Target Compound	C27H50NO7P	530.32521	Negative	M-H	1	2.7
LPE 22:4	Target Compound	C27H48NO7P	528.30956	Negative	M-H	1	2.68
LPE 22:5	Target Compound	C27H46NO7P	526.29391	Negative	M-H	1	2.55
LPE 22:6	Target Compound	C27H44NO7P	524.27826	Negative	M-H	1	2.51
LPE 24:0	Target Compound	C29H60NO7P	564.40346	Negative	M-H	1	3.29
LPE O-16:1	Target Compound	C21H44NO6P	436.28335	Negative	M-H	1	2.73
LPE O-18:1	Target Compound	C23H48NO6P	464.31465	Negative	M-H	1	2.95
LPE O-18:2	Target Compound	C23H46NO6P	462.299	Negative	M-H	1	2.76
LPE O-20:1	Target Compound	C25H52NO6P	492.34595	Negative	M-H	1	3.21
LPG 16:0	Target Compound	C22H45O9P	483.27284	Negative	M-H	1	2.53
LPG 17:1	Internal Standard	C23H45O9P	495.27284	Negative	M-H	1	2.49
LPG 18:0	Target Compound	C24H49O9P	511.30414	Negative	M-H	1	2.69
LPG 18:1	Target Compound	C24H47O9P	509.28849	Negative	M-H	1	2.57
LPG 18:2	Target Compound	C24H45O9P	507.27284	Negative	M-H	1	2.47
LPI 16:0	Target Compound	C25H49O12P	571.28889	Negative	M-H	1	2.5
LPI 17:1	Internal Standard	C26H49O12P	583.28889	Negative	M-H	1	2.46
LPI 18:0	Target Compound	C27H53O12P	599.32019	Negative	M-H	1	2.65
LPI 18:1	Target Compound	C27H51O12P	597.30454	Negative	M-H	1	2.53
LPI 18:2	Target Compound	C27H49O12P	595.28889	Negative	M-H	1	2.44
LPI 20:3	Target Compound	C29H51O12P	621.30454	Negative	M-H	1	2.49
LPI 20:4	Target Compound	C29H49O12P	619.28889	Negative	M-H	1	2.43
LPS 18:0	Target Compound	C24H48NO9P	524.29939	Negative	M-H	1	2.62
LPS 18:1	Target Compound	C24H46NO9P	522.28374	Negative	M-H	1	2.45
LPS 20:4	Target Compound	C26H44NO9P	544.26809	Negative	M-H	1	2.37
LPS 22:6	Target Compound	C28H44NO9P	568.26809	Negative	M-H	1	2.35
NeuAcHex2Cer 34:1;O2	Target Compound	C57H104N2O21	1151.70588	Negative	M-H	1	3.69
PA 34:1	Target Compound	C37H71O8P	673.48138	Negative	M-H	1	4.61
PA 36:1	Target Compound	C39H75O8P	701.51268	Negative	M-H	1	5.14
PA 36:2	Target Compound	C39H73O8P	699.49703	Negative	M-H	1	4.7
PC 15:0/18:1-d7	Internal Standard	D7C41H73NO8P	797.60429	Negative	M+HCO2	1	4.47
PC 34:1;O	Target Compound	C42H80NO9P	818.55527	Negative	M+HCO2	1	3.27
PC 34:2;O2	Target Compound	C42H80NO10P	834.55019	Negative	M+HCO2	1	3.23
PC 36:1;O	Target Compound	C44H84NO9P	846.58657	Negative	M+HCO2	1	3.6
PC 36:2;O	Target Compound	C44H82NO9P	844.57092	Negative	M+HCO2	1	3.38
PC 36:3;O2	Target Compound	C44H80NO10P	858.55019	Negative	M+HCO2	1	2.89
PC 36:3;O	Target Compound	C44H80NO9P	842.55527	Negative	M+HCO2	1	3.5
PC 38:5;O	Target Compound	C46H80NO9P	866.55527	Negative	M+HCO2	1	3.28

PC O-18:0/18:1-d9	Internal Standard	D9C44H77NO7P	825.66888	Negative	M+HCO2	1	5.51
PE 15:0/18:1-d7	Internal Standard	D7C38H67NO8P	709.55187	Negative	M-H	1	4.63
PE 32:0	Target Compound	C37H74NO8P	690.50793	Negative	M-H	1	4.82
PE 32:1	Target Compound	C37H72NO8P	688.49228	Negative	M-H	1	4.42
PE 34:1	Target Compound	C39H76NO8P	716.52358	Negative	M-H	1	4.9
PE 34:2	Target Compound	C39H74NO8P	714.50793	Negative	M-H	1	4.51
PE 34:3	Target Compound	C39H72NO8P	712.49228	Negative	M-H	1	4.16
PE 35:2	Target Compound	C40H76NO8P	728.52358	Negative	M-H	1	4.74
PE 36:1	Target Compound	C41H80NO8P	744.55488	Negative	M-H	1	5.41
PE 36:2	Target Compound	C41H78NO8P	742.53923	Negative	M-H	1	4.99
PE 36:3	Target Compound	C41H76NO8P	740.52358	Negative	M-H	1	4.59
PE 36:4	Target Compound	C41H74NO8P	738.50793	Negative	M-H	1	4.43
PE 36:5	Target Compound	C41H72NO8P	736.49228	Negative	M-H	1	4.1
PE 37:4	Target Compound	C42H76NO8P	752.52358	Negative	M-H	1	4.66
PE 38:2	Target Compound	C43H82NO8P	770.57053	Negative	M-H	1	5.52
PE 38:4	Target Compound	C43H78NO8P	766.53923	Negative	M-H	1	4.92
PE 38:6	Target Compound	C43H74NO8P	762.50793	Negative	M-H	1	4.27
PE 40:1	Target Compound	C45H88NO8P	800.61748	Negative	M-H	1	6.4
PE 40:3	Target Compound	C45H84NO8P	796.58618	Negative	M-H	1	5.45
PE 40:4	Target Compound	C45H82NO8P	794.57053	Negative	M-H	1	5.16
PE 40:5	Target Compound	C45H80NO8P	792.55488	Negative	M-H	1	4.89
PE 40:6	Target Compound	C45H78NO8P	790.53923	Negative	M-H	1	4.74
PE 40:7	Target Compound	C45H76NO8P	788.52358	Negative	M-H	1	4.36
PE 40:8	Target Compound	C45H74NO8P	786.50793	Negative	M-H	1	4.07
PE 41:1	Target Compound	C46H90NO8P	814.63313	Negative	M-H	1	6.6
PE 42:1	Target Compound	C47H92NO8P	828.64878	Negative	M-H	1	6.89
PE 42:10	Target Compound	C47H74NO8P	810.50793	Negative	M-H	1	3.95
PE 42:7	Target Compound	C47H80NO8P	816.55488	Negative	M-H	1	4.81
PE 42:8	Target Compound	C47H78NO8P	814.53923	Negative	M-H	1	4.23
PE 44:10	Target Compound	C49H78NO8P	838.53923	Negative	M-H	1	4.12
PE 44:11	Target Compound	C49H76NO8P	836.52358	Negative	M-H	1	3.83
PE O-16:1_16:0	Target Compound	C37H74NO7P	674.51301	Negative	M-H	1	5.15
PE O-16:1_18:1	Target Compound	C39H76NO7P	700.52866	Negative	M-H	1	5.26
PE O-16:1_18:2	Target Compound	C39H74NO7P	698.51301	Negative	M-H	1	4.8
PE O-16:1_20:4	Target Compound	C41H74NO7P	722.51301	Negative	M-H	1	4.71
PE O-16:1_22:6	Target Compound	C43H74NO7P	746.51301	Negative	M-H	1	4.54
PE O-18:1_16:0	Target Compound	C39H78NO7P	702.54431	Negative	M-H	1	5.67
PE O-18:1_18:1	Target Compound	C41H80NO7P	728.55996	Negative	M-H	1	5.77
PE O-18:1_18:2	Target Compound	C41H78NO7P	726.54431	Negative	M-H	1	5.32
PE O-18:1_20:4	Target Compound	C43H78NO7P	750.54431	Negative	M-H	1	5.24
PE O-18:1_22:6	Target Compound	C45H78NO7P	774.54431	Negative	M-H	1	5.06
PE O-18:2_14:0	Target Compound	C37H72NO7P	672.49736	Negative	M-H	1	4.74
PE O-18:2_18:2	Target Compound	C41H76NO7P	724.52866	Negative	M-H	1	4.89
PE O-18:2_20:4	Target Compound	C43H76NO7P	748.52866	Negative	M-H	1	4.81
PE O-35:2	Target Compound	C40H78NO7P	714.54431	Negative	M-H	1	5.52
PE O-35:3	Target Compound	C40H76NO7P	712.52866	Negative	M-H	1	5.05
PE O-36:1	Target Compound	C41H82NO7P	730.57561	Negative	M-H	1	5.84
PE O-36:6	Target Compound	C41H72NO7P	720.49736	Negative	M-H	1	4.35
PE O-38:1	Target Compound	C43H86NO7P	758.60691	Negative	M-H	1	6.24
PE O-38:2	Target Compound	C43H84NO7P	756.59126	Negative	M-H	1	6.3
PE O-38:3	Target Compound	C43H82NO7P	754.57561	Negative	M-H	1	5.9
PE O-38:4	Target Compound	C43H80NO7P	752.55996	Negative	M-H	1	5.45
PE O-40:2	Target Compound	C45H88NO7P	784.62256	Negative	M-H	1	6.85
PE O-40:3	Target Compound	C45H86NO7P	782.60691	Negative	M-H	1	6.34
PE O-40:5	Target Compound	C45H82NO7P	778.57561	Negative	M-H	1	5.79
PE O-40:6	Target Compound	C45H80NO7P	776.55996	Negative	M-H	1	5.24
PE O-40:8	Target Compound	C45H76NO7P	772.52866	Negative	M-H	1	4.58
PE O-42:3	Target Compound	C47H90NO7P	810.63821	Negative	M-H	1	6.91
PE O-42:5	Target Compound	C47H86NO7P	806.60691	Negative	M-H	1	6.38
PE O-42:6	Target Compound	C47H84NO7P	804.59126	Negative	M-H	1	5.76
PG 15:0/18:1-d7	Internal Standard	D7C39H68O10P	740.54645	Negative	M-H	1	4
PG 30:0	Target Compound	C36H71O10P	693.47121	Negative	M-H	1	3.7
PG 32:0	Target Compound	C38H75O10P	721.50251	Negative	M-H	1	4.07
PG 32:1	Target Compound	C38H73O10P	719.48686	Negative	M-H	1	3.87
PG 34:1	Target Compound	C40H77O10P	747.51816	Negative	M-H	1	4.52
PG 34:2	Target Compound	C40H75O10P	745.50251	Negative	M-H	1	3.95
PG 36:1	Target Compound	C42H81O10P	775.54946	Negative	M-H	1	5.02
PG 36:2	Target Compound	C42H79O10P	773.53381	Negative	M-H	1	4.6
PG 36:3	Target Compound	C42H77O10P	771.51816	Negative	M-H	1	4.23

PG 36:4	Target Compound	C42H75O10P	769.50251	Negative	M-H	1	3.84
PG 38:4	Target Compound	C44H79O10P	797.53381	Negative	M-H	1	4.23
PG 38:5	Target Compound	C44H77O10P	795.51816	Negative	M-H	1	3.76
PG 38:6	Target Compound	C44H75O10P	793.50251	Negative	M-H	1	3.5
PG 40:6	Target Compound	C46H79O10P	821.53381	Negative	M-H	1	3.95
PG 40:7	Target Compound	C46H77O10P	819.51816	Negative	M-H	1	3.65
PG 40:8	Target Compound	C46H75O10P	817.50251	Negative	M-H	1	3.46
PI 15:0/18:1-d7	Internal Standard	D7C42H72O13P	828.56249	Negative	M-H	1	3.94
PI 32:0	Target Compound	C41H79O13P	809.51855	Negative	M-H	1	4.04
PI 32:1	Target Compound	C41H77O13P	807.5029	Negative	M-H	1	3.78
PI 33:1	Target Compound	C42H79O13P	821.51855	Negative	M-H	1	3.95
PI 34:0	Target Compound	C43H83O13P	837.54985	Negative	M-H	1	4.43
PI 34:1	Target Compound	C43H81O13P	835.5342	Negative	M-H	1	4.12
PI 34:2	Target Compound	C43H79O13P	833.51855	Negative	M-H	1	3.83
PI 35:1	Target Compound	C44H83O13P	849.54985	Negative	M-H	1	4.31
PI 35:2	Target Compound	C44H81O13P	847.5342	Negative	M-H	1	4.01
PI 36:1	Target Compound	C45H85O13P	863.5655	Negative	M-H	1	4.54
PI 36:2	Target Compound	C45H83O13P	861.54985	Negative	M-H	1	4.21
PI 36:3	Target Compound	C45H81O13P	859.5342	Negative	M-H	1	3.93
PI 36:4	Target Compound	C45H79O13P	857.51855	Negative	M-H	1	3.79
PI 36:5	Target Compound	C45H77O13P	855.5029	Negative	M-H	1	3.55
PI 37:4	Target Compound	C46H81O13P	871.5342	Negative	M-H	1	3.97
PI 38:3	Target Compound	C47H85O13P	887.5655	Negative	M-H	1	4.31
PI 38:4	Target Compound	C47H83O13P	885.54985	Negative	M-H	1	4.17
PI 38:5	Target Compound	C47H81O13P	883.5342	Negative	M-H	1	3.87
PI 38:6	Target Compound	C47H79O13P	881.51855	Negative	M-H	1	3.68
PI 40:5	Target Compound	C49H85O13P	911.5655	Negative	M-H	1	4.14
PI 40:6	Target Compound	C49H83O13P	909.54985	Negative	M-H	1	4.02
PI O-18:1_20:4	Target Compound	C47H83O12P	869.55494	Negative	M-H	1	4.12
PS 15:0/18:1-d7	Internal Standard	D7C39H67NO10P	753.54169	Negative	M-H	1	3.99
PS 34:1	Target Compound	C40 H76 N O10 P	760.51341	Negative	M-H	1	4.17
PS 36:1	Target Compound	C42H80NO10P	788.54471	Negative	M-H	1	4.59
PS 36:2	Target Compound	C42H78NO10P	786.52906	Negative	M-H	1	4.27
PS 36:4	Target Compound	C42 H74 N O10 P	782.49776	Negative	M-H	1	3.84
PS 38:1	Target Compound	C44 H84 N O10 P	816.57601	Negative	M-H	1	5.02
PS 38:2	Target Compound	C44H82NO10P	814.56036	Negative	M-H	1	4.71
PS 38:3	Target Compound	C44 H80 N O10 P	812.54471	Negative	M-H	1	4.39
PS 38:4	Target Compound	C44 H78 N O10 P	810.52906	Negative	M-H	1	4.22
PS 38:5	Target Compound	C44 H76 N O10 P	808.5134	Negative	M-H	1	3.92
PS 38:6	Target Compound	C44H74NO10P	806.49776	Negative	M-H	1	3.72
PS 40:1	Target Compound	C46H88NO10P	844.60731	Negative	M-H	1	5.64
PS 40:2	Target Compound	C46H86NO10P	842.59166	Negative	M-H	1	5.1
PS 40:4	Target Compound	C46 H82 N O10 P	838.56036	Negative	M-H	1	4.5
PS 40:5	Target Compound	C46 H80 N O10 P	836.5447	Negative	M-H	1	4.36
PS 40:6	Target Compound	C46 H78 N O10 P	834.52906	Negative	M-H	1	4.06
PS 40:7	Target Compound	C46 H76 N O10 P	832.51341	Negative	M-H	1	3.77
PS 42:1	Target Compound	C48H92NO10P	872.63861	Negative	M-H	1	6.4
PS 42:2	Target Compound	C48H90NO10P	870.62296	Negative	M-H	1	5.69
PS 44:10	Target Compound	C50 H78 N O10 P	882.52906	Negative	M-H	1	3.66
PS 44:12	Target Compound	C50 H74 N O10 P	878.49776	Negative	M-H	1	3.31
SHexCer 36:1;O2	Target Compound	C42H81NO11S	806.54576	Negative	M-H	1	4.16
SHexCer 36:1;O3	Target Compound	C42H81NO12S	822.54067	Negative	M-H	1	4.05
SHexCer 38:1;O2	Target Compound	C44H85NO11S	834.57706	Negative	M-H	1	4.6
SHexCer 38:1;O3	Target Compound	C44H85NO12S	850.57197	Negative	M-H	1	4.48
SHexCer 40:1;O2	Target Compound	C46H89NO11S	862.60836	Negative	M-H	1	5.07
SHexCer 40:1;O3	Target Compound	C46H89NO12S	878.60327	Negative	M-H	1	4.96
SHexCer 40:2;O2	Target Compound	C46H87NO11S	860.59271	Negative	M-H	1	4.61
SHexCer 41:1;O3	Target Compound	C47H91NO12S	892.61892	Negative	M-H	1	5.24
SHexCer 42:0;O2	Target Compound	C48H95NO11S	892.65531	Negative	M-H	1	5.9
SHexCer 42:1;O2	Target Compound	C48H93NO11S	890.63966	Negative	M-H	1	5.62
SHexCer 42:2;O2	Target Compound	C48H91NO11S	888.62401	Negative	M-H	1	5.05
SHexCer 42:2;O3	Target Compound	C48H91NO12S	904.61892	Negative	M-H	1	5
SHexCer 42:3;O2	Target Compound	C48H89NO11S	886.60836	Negative	M-H	1	4.64
SHexCer 44:2;O2	Target Compound	C50H95NO11S	916.65531	Negative	M-H	1	5.6
SM 18:1;O2/18:1-d9	Internal Standard	D9C41H72N2O6P	782.63792	Negative	M+HCO2	1	4.35
ST 19:2;O2;S	Target Compound	C19H28O5S	367.15847	Negative	M-H	1	1.72
ST 19:2;O3;S	Target Compound	C19H28O6S	383.15338	Negative	M-H	1	1.11
ST 27:1;O;S	Target Compound	C27H46O4S	465.3044	Negative	M-H	1	2.88
Thyroxin	Target Compound	C15H11I4NO4	775.6794	Negative	M-H	1	1.88

Metabolomics: LC-MS/MS Instrumentation and Analysis

Instrumentation

HPLC:	Thermo Vanquish Horizon
MS:	Thermo Orbitrap Exploris 480
Mode	H-ESI - positive and negative electrospray ionization
Column	SeQuant ZIC-HILIC 2.1 x 100 mm 3.5 µm (Merck)
Pre-column	SeQuant ZIC-HILIC 2.1 x 20 mm Guard Kit (Merck)

Analysis

Data Acquisition	Xcalibur 4.4
Data Evaluation	TraceFinder 5.1
Method	Scan + $ddMS^2$

LC parameters

Solvents

A	Water + 0.1% formic acid
B	Acetonitrile + 0.1% formic acid

Gradient Elution

Time [min]	A [%]	B [%]	Flowrate [mL/min]
0.00	5.00	95.00	0.300
1.50	5.00	95.00	0.300
12.00	60.00	40.00	0.300
14.00	60.00	40.00	0.300
14.20	75.00	25.00	0.300
17.00	75.00	25.00	0.300
19.00	5.00	95.00	0.500
26.00	5.00	95.00	0.500

Injection Volume

Positive Ionization Mode	3 µL
Negative Ionization Mode	5 µL

Positive Ionization

MS parameters

Ion Source

Parameters	Value
Ion Source Type:	H-ESI
Spray Voltage:	Static
Positive Ion (V):	3400
Negative Ion (V):	3000
Gas Mode:	Static
Sheath Gas (Arb):	25
Aux Gas (Arb):	7
Sweep Gas (Arb):	0
Ion Transfer Tube Temp (°C):	320
Vaporizer Temp (°C):	180

MS Global Settings

Parameters	Value
Infusion Mode:	Liquid Chromatography
Expected LC Peak Width (s):	6
Advanced Peak Determination:	TRUE
Mild Trapping:	TRUE
Default Charge State:	1
Enable Xcalibur AcquireX method configurations	TRUE

Negative Ionization

MS parameters

Ion Source

Parameters	Value
Ion Source Type:	H-ESI
Spray Voltage:	Static
Positive Ion (V):	3400
Negative Ion (V):	3000
Gas Mode:	Static
Sheath Gas (Arb):	25
Aux Gas (Arb):	7
Sweep Gas (Arb):	0
Ion Transfer Tube Temp (°C):	320
Vaporizer Temp (°C):	180

MS Global Settings

Parameters	Value
Infusion Mode:	Liquid Chromatography
Expected LC Peak Width (s):	6
Advanced Peak Determination:	TRUE
Mild Trapping:	TRUE
Default Charge State:	1
Enable Xcalibur AcquireX method	TRUE

Internal Mass Calibration: RunStart EASY-IC™

Settings for Full Scan
Orbitrap Resolution: 120000
Scan Range (m/z): 70-700
RF Lens (%): 20.0
AGC Target: Custom
Normalized AGC Target (%): 100.0
Maximum Injection Time Mode: Auto
Microscans: 1.0
Data Type: Profile
Polarity: Positive
Source Fragmentation: Disabled

Filters
Intensity
Filter Type: Intensity Threshold
Intensity Threshold: 5000.0

Dynamic Exclusion
Dynamic Exclusion Mode: Custom
Exclude after n times: 1.0
Exclusion duration (s): 5.0
Mass Tolerance: ppm
Low 10.0
High 10.0
Exclude isotopes: TRUE
Perform dependent scan on single charge state FALSE

Targeted Mass
Mass List
Mass List Type: m/z
Time Mode: Start/End Time
Include Intensity Threshold: TRUE
Add Mass List Targets Determined by Xcalibur A TRUE

Compound	m/z	t start (min)	t stop (min)	Intensity Threshold
MRFA (Placeholder)	524.265	0.0	26.0	0.0

Mass Tolerance: ppm
Low: 10.0
High: 10.0
Set Collision Energy per Compound: FALSE
Perform dependent scan on most intense ion if n TRUE

Targeted Mass Exclusion
Mass List
Mass List Type: m/z
Time Mode: Start/End Time
Include Intensity Threshold: TRUE
Add Mass List Targets Determined by Xcalibur A TRUE

Compound	m/z	t start (min)	t stop (min)	Intensity Threshold
MRFA (Placeholder)	524.265	0.0	26.0	1E+20

Mass Tolerance: ppm
Low: 10.0
High: 10.0

Apex Detection
Desired Apex Window (%): 30.0

Data Dependent
Data Dependent Mode: Cycle Time
Time between Master Scans (sec): 0.6

Settings for ddMS^2
Multiplex Ions: FALSE

Internal Mass Calibration: RunStart EASY-IC™

Settings for Full Scan
Orbitrap Resolution: 120000
Scan Range (m/z): 59-590
RF Lens (%): 30.0
AGC Target: Custom
Normalized AGC Target (%): 100.0
Maximum Injection Time Mode: Auto
Microscans: 1.0
Data Type: Profile
Polarity: Negative
Source Fragmentation: Disabled

Filters
Intensity
Filter Type: Intensity Threshold
Intensity Threshold: 5000.0

Dynamic Exclusion
Dynamic Exclusion Mode: Custom
Exclude after n times: 1.0
Exclusion duration (s): 5.0
Mass Tolerance: ppm
Low 10.0
High 10.0
Exclude isotopes: TRUE
Perform dependent scan on single FALSE

Targeted Mass
Mass List
Mass List Type: m/z
Time Mode: Start/End Time
Include Intensity Threshold: TRUE
Add Mass List Targets Determined TRUE

Compound	m/z	t start (min)	t stop (min)	Intensity Threshold
MRFA (Placeholder)	524.265	0.0	26.0	0.0

Mass Tolerance: ppm
Low: 10.0
High: 10.0
Set Collision Energy per Compour FALSE
Perform dependent scan on most TRUE

Targeted Mass Exclusion
Mass List
Mass List Type: m/z
Time Mode: Start/End Time
Include Intensity Threshold: TRUE
Add Mass List Targets Determined TRUE

Compound	m/z	t start (min)	t stop (min)	Intensity Threshold
MRFA (Placeholder)	524.265	0.0	26.0	1E+20

Mass Tolerance: ppm
Low: 10.0
High: 10.0

Apex Detection
Desired Apex Window (%): 30.0

Data Dependent
Data Dependent Mode: Cycle Time
Time between Master Scans (sec) 0.6

Settings for ddMS^2
Multiplex Ions: FALSE

Isolation Window (m/z):	1.0	Isolation Window (m/z):	1.0
Isolation Offset:	Off	Isolation Offset:	Off
Collision Energy Mode:	Stepped	Collision Energy Mode:	Stepped
Collision Energy Type:	Absolute	Collision Energy Type:	Absolute
HCD Collision Energies (V):	15,30,60	HCD Collision Energies (V):	15,30,60
Orbitrap Resolution:	15000.0	Orbitrap Resolution:	15000.0
Scan Range Mode:	Auto	Scan Range Mode:	Auto
AGC Target:	Custom	AGC Target:	Custom
Normalized AGC Target (%):	10.0	Normalized AGC Target (%):	10.0
Maximum Injection Time Mode:	Auto	Maximum Injection Time Mode:	Auto
Microscans:	1.0	Microscans:	1.0
Data Type:	Profile	Data Type:	Profile

Internal Standard

**Isotopically labeled internal standard**	**Vendor**	**Category**	**Concentration of standar**	**Volume of working soluti**	**Sample Volume [µL]**	**Concentration related to sample [ng/mL]**
Cholic acid-2,2,4,4-d4	Cambridge Isotope Laboratorie:	A	66.7	75	10	500
D-Fructose-U13C6	Cambridge Isotope Laboratorie:	A	66.7	75	10	500
Glycine-d5	Sigma Aldrich	A	66.7	75	10	500
Hypoxanthine-13C5	Cambridge Isotope Laboratorie:	B	6.7	75	10	50
L-Carnitine-d9	Cambridge Isotope Laboratorie:	A	66.7	75	10	500
L-Isoleucine-d10	Cambridge Isotope Laboratorie:	A	66.7	75	10	500
L-Leucine-d10	Cambridge Isotope Laboratorie:	A	66.7	75	10	500
L-Lysine-d9	Cambridge Isotope Laboratorie:	A	66.7	75	10	500
Succinic acid-2,2,3,3-d4	Sigma-Aldrich	A	66.7	75	10	500

*Cambridge Isotopes Laboratories Tewksbury, MA, USA
*Sigma Aldrich St. Louis, MO, USA

Acceptance criteria

The following analytes were excluded from data evaluation:
- Analytes showing a mass error > 5 ppm
- Analytes showing an isotope score < 40 (10 ppm, 90% intensity)
- Analytes without identity confirmation via spectral library matching or reference substance measurements
- Analytes containing only missing values in QC samples and study samples
- Analytes containing > 10% missing values in QC samples and study samples
- Analytes showing > 30% area RSD in QC samples

Acquisition List

Compound Name	Compound Type	Compound Formula	m/z	Polarity	Adduct	Charge State	Retention Time
5-Hydroxylysine	TargetCompound	C6H14N2O3	163.1077	Positive	M+H	1	11.74
Acetamidobutanoic acid	TargetCompound	C6H11NO3	146.0812	Positive	M+H	1	1.67
Acetyllysine	TargetCompound	C8H16N2O3	189.1234	Positive	M+H	1	7.85
Adenosine	TargetCompound	C10H13N5O4	268.104	Positive	M+H	1	6.04
Alanine	TargetCompound	C3H7NO2	90.055	Positive	M+H	1	8
alpha-Aminoadipic acid	TargetCompound	C6H11NO4	162.0761	Positive	M+H	1	7.69
Aminobutyric acid	TargetCompound	C4H9NO2	104.0706	Positive	M+H	1	7.52
Aminooctanoic acid	TargetCompound	C8H17NO2	160.1332	Positive	M+H	1	3.85
Anserine	TargetCompound	C10H16N4O3	241.1295	Positive	M+H	1	12.48
Arginine	TargetCompound	C6H14N4O2	175.119	Positive	M+H	1	11.2
Asparagine	TargetCompound	C4H8N2O3	133.0608	Positive	M+H	1	8.77
Aspartic acid	TargetCompound	C4H7NO4	134.0448	Positive	M+H	1	8.46
Betaine	TargetCompound	C5H11NO2	118.0863	Positive	M+H	1	7.6
CAR 2:0	TargetCompound	C9H17NO4	204.123	Positive	M+H	1	3.27
CAR 3:0	TargetCompound	C10H19NO4	218.1387	Positive	M+H	1	2.73
CAR 4:0	TargetCompound	C11H21NO4	232.1543	Positive	M+H	1	2.31
CAR 5:0	TargetCompound	C12H23NO4	246.17	Positive	M+H	1	2.11
CAR 6:0	TargetCompound	C13H25NO4	260.1856	Positive	M+H	1	2.01
CAR 8:0	TargetCompound	C15H29NO4	288.2169	Positive	M+H	1	2.11
CAR DC3:0;2Me	TargetCompound	C11H19NO6	262.1285	Positive	M+H	1	6.03
CAR DC5:0	TargetCompound	C12H21NO6	276.1442	Positive	M+H	1	7.35
CAR DC5:0;3Me	TargetCompound	C13H23NO6	290.1598	Positive	M+H	1	5.71
Carnitine	TargetCompound	C7H15NO3	162.1125	Positive	M+H	1	7.02
Carnitine-d9	InternalStandard	C7H6NO3D9	171.169	Positive	M+H	1	7.02
Carnosine	TargetCompound	C9H14N4O3	227.1139	Positive	M+H	1	11.5
Choline	TargetCompound	C5H14NO	104.107	Positive	M+	1	6.31
cis-3-Hydroxyproline	TargetCompound	C5H9NO3	132.0655	Positive	M+H	1	8.15

cis-4-Hydroxyproline	TargetCompound	C5H9NO3		132.0655	Positive	M+H	1	8.13
Citrulline	TargetCompound	C6H13N3O3		176.103	Positive	M+H	1	8.97
Creatine	TargetCompound	C4H9N3O2		132.0768	Positive	M+H	1	7.68
Creatinine	TargetCompound	C4H7N3O		114.0662	Positive	M+H	1	7.22
Cystathionine	TargetCompound	C7H14N2O4S		223.0747	Positive	M+H	1	10.41
Cysteine	TargetCompound	C3H7NO2S		122.027	Positive	M+H	1	7.75
Cysteine-S-sulfate	TargetCompound	C3H7NO5S2		201.9838	Positive	M+H	1	7.25
Cystine	TargetCompound	C6H12N2O4S2		241.0311	Positive	M+H	1	10.39
Cytidine	TargetCompound	C9H13N3O5		244.0928	Positive	M+H	1	8.2
Dimethylarginine	TargetCompound	C8H18N4O2		203.1503	Positive	M+H	1	10.45
Dimethylglycine	TargetCompound	C4H9NO2		104.0706	Positive	M+H	1	7.52
DOPA	TargetCompound	C9H11NO4		198.0761	Positive	M+H	1	7.64
Dopamine	TargetCompound	C8H11NO2		154.0863	Positive	M+H	1	7.42
Ergothioneine	TargetCompound	C9H15N3O2S		230.0958	Positive	M+H	1	8.83
Glutamic acid	TargetCompound	C5H9NO4		148.0604	Positive	M+H	1	7.98
Glutamic acid-d5	InternalStandard	C5H4NO4D5		153.0918	Positive	M+H	1	7.98
Glutamine	TargetCompound	C5H10N2O3		147.0764	Positive	M+H	1	8.62
Glycine	TargetCompound	C2H5NO2		76.0393	Positive	M+H	1	8.65
Glycine-d2	InternalStandard	C2H3NO2D2		78.0519	Positive	M+H	1	8.65
Guanidinobutanoic acid	TargetCompound	C5H11N3O2		146.0924	Positive	M+H	1	6.86
Guanidinosuccinic acid	TargetCompound	C5H9N3O4		176.0666	Positive	M+H	1	6.42
Guanine	TargetCompound	C5H5N5O		152.0567	Positive	M+H	1	6.92
Histamine	TargetCompound	C5H9N3		112.0869	Positive	M+H	1	13.06
Histidine	TargetCompound	C6H9N3O2		156.0768	Positive	M+H	1	10.93
Homoarginine	TargetCompound	C7H16N4O2		189.1346	Positive	M+H	1	11.17
Homocysteine	TargetCompound	C4H9NO2S		136.0427	Positive	M+H	1	7.25
Hydroxybenzaldehyde	TargetCompound	C7H6O2		123.0441	Positive	M+H	1	7.21
Hypotaurine	TargetCompound	C2H7NO2S		110.027	Positive	M+H	1	8.52
Hypoxanthine	TargetCompound	C5H4N4O		137.0458	Positive	M+H	1	5.19
Hypoxanthine-13C5	InternalStandard	[13]C5H4N4O		142.0626	Positive	M+H	1	5.19
Indoleacrylic acid	TargetCompound	C11H9NO2		188.0706	Positive	M+H	1	6.46
Indolepropionic acid	TargetCompound	C11H11NO2		190.0863	Positive	M+H	1	6.17
Inosine	TargetCompound	C10H12N4O5		269.0881	Positive	M+H	1	5.98
Isoleucine	TargetCompound	C6H13NO2		132.1019	Positive	M+H	1	6.25
Kynurenic acid	TargetCompound	C10H7NO3		190.0499	Positive	M+H	1	4.41
Kynurenine	TargetCompound	C10H12N2O3		209.0921	Positive	M+H	1	6.3
Leucine	TargetCompound	C6H13NO2		132.1019	Positive	M+H	1	6.25
Leucine-d10	InternalStandard	C6H3NO2D10		142.1647	Positive	M+H	1	6.21
Leu-Pro	TargetCompound	C11H20N2O3		229.1547	Positive	M+H	1	4.87
Lysine	TargetCompound	C6H14N2O2		147.1128	Positive	M+H	1	11.33
Lysine-d9	InternalStandard	C6H5N2O2D9		156.1693	Positive	M+H	1	11.34
Methionine	TargetCompound	C5H11NO2S		150.0583	Positive	M+H	1	6.76
Methionine sulfoxide	TargetCompound	C5H11NO3S		166.0532	Positive	M+H	1	8.73
Methyladenosine	TargetCompound	C11 H15 N5 O4		282.1197	Positive	M+H	1	7.65
Methylhistamine	TargetCompound	C6H11N3		126.1026	Positive	M+H	1	15.44
Methylhistidine	TargetCompound	C7H11N3O2		170.0924	Positive	M+H	1	10.97
Methylimidazoleacetic acid	TargetCompound	C6H8N2O2		141.0659	Positive	M+H	1	6.58
Methylxanthine	TargetCompound	C6H6N4O2		167.0564	Positive	M+H	1	2.59
N4-Acetylcytidine	TargetCompound	C11 H15 N3 O6		286.1034	Positive	M+H	1	6.37
N-6-Trimethyllysine	TargetCompound	C9H20N2O2		189.1598	Positive	M+H	1	11.61
N-Acetylarginine	TargetCompound	C8H16N4O3		217.1295	Positive	M+H	1	7.59
N-Acetylcarnosine	TargetCompound	C11H16N4O4	<multiple>		Positive	M+H	1	8
N-Acetylornithine	TargetCompound	C7H14N2O3		175.1077	Positive	M+H	1	7.52
N-Acetylserine	TargetCompound	C5H9NO4		148.0604	Positive	M+H	1	7.2
Niacinamide	TargetCompound	C6H6N2O		123.0553	Positive	M+H	1	2.33
O-Acetylserine	TargetCompound	C5H9NO4		148.0604	Positive	M+H	1	7.2
Ornithine	TargetCompound	C5H12N2O2		133.0972	Positive	M+H	1	11.44
Pantothenic acid	TargetCompound	C9H17NO5		220.118	Positive	M+H	1	2.53
Paraxanthine / Theobromine / Theophylline	TargetCompound	C7H8N4O2		181.072	Positive	M+H	1	1.76
Phenylalanine	TargetCompound	C9H11NO2		166.0863	Positive	M+H	1	6.17
Phenylethylamine	TargetCompound	C8H11N		122.0964	Positive	M+H	1	2.3
Phenylglycine	TargetCompound	C8H9NO2		152.0706	Positive	M+H	1	5.54
Pipecolic acid	TargetCompound	C6H11NO2		130.0863	Positive	M+H	1	11.34
Proline	TargetCompound	C5H9NO2		116.0706	Positive	M+H	1	7.61
Proline betaine	TargetCompound	C7H13NO2		144.1019	Positive	M+H	1	7
Pyroglutamic acid	TargetCompound	C5H7NO3		130.0499	Positive	M+H	1	8.49
Sarcosine	TargetCompound	C3H7NO2		90.055	Positive	M+H	1	7.89
Serine	TargetCompound	C3H7NO3		106.0499	Positive	M+H	1	8.77
Serotonin	TargetCompound	C10H12N2O		177.1022	Positive	M+H	1	7.1

Taurine	TargetCompound	C2H7NO3S	126.0219	Positive	M+H	1	8.14
Threonine	TargetCompound	C4H9NO3	120.0655	Positive	M+H	1	8.31
Trigonelline	TargetCompound	C7H7NO2	138.055	Positive	M+H	1	7.03
Trimethylamine N-oxide	TargetCompound	C3H9NO	76.0757	Positive	M+H	1	7.1
Tryptophan	TargetCompound	C11H12N2O2	205.0972	Positive	M+H	1	6.46
Tyrosine	TargetCompound	C9H11NO3	182.0812	Positive	M+H	1	7.22
Uracil	TargetCompound	C4H4N2O2	113.0346	Positive	M+H	1	5.16
Urocanic acid	TargetCompound	C6H6N2O2	139.0502	Positive	M+H	1	6.3
Valine	TargetCompound	C5H11NO2	118.0863	Positive	M+H	1	6.92
Xanthine	TargetCompound	C5H4N4O2	153.0407	Positive	M+H	1	5.1
Acetamidobutanoic acid	TargetCompound	C6H11NO3	144.0666	Negative	M-H	1	1.5
Allantoin	TargetCompound	C4H6N4O3	157.0367	Negative	M-H	1	6.67
alpha-N-Phenylacetylglutamine	TargetCompound	C13H16N2O4	263.1037	Negative	M-H	1	1.61
Aminohippuric acid	TargetCompound	C9H10N2O3	193.0619	Negative	M-H	1	1.92
AMP	TargetCompound	C10H14N5O7P	346.0558	Negative	M-H	1	9
Ascorbic acid sulfate	TargetCompound	C6H8O9S	254.9816	Negative	M-H	1	5.47
BA 24:1;O3;G	TargetCompound	C26H43NO4	432.3119	Negative	M-H	1	1.27
BA 24:1;O3;G;S	TargetCompound	C26H43NO7S	512.2688	Negative	M-H	1	4.7
BA 24:1;O3;T	TargetCompound	C26H45NO5S	482.2946	Negative	M-H	1	4.62
BA 24:1;O4	TargetCompound	C24H40O4	391.2854	Negative	M-H	1	1
BA 24:1;O4;G	TargetCompound	C26H43NO5	448.3069	Negative	M-H	1	1.57
BA 24:1;O4;T	TargetCompound	C26H45NO6S	498.2895	Negative	M-H	1	4.99
BA 24:1;O5	TargetCompound	C24H40O5	407.2803	Negative	M-H	1	1.2
BA 24:1;O5;G	TargetCompound	C26H43NO6	464.3018	Negative	M-H	1	3.77
BA 24:1;O5;T	TargetCompound	C26H45NO7S	514.2844	Negative	M-H	1	5.46
Bilirubin	TargetCompound	C33H36N4O6	583.2562	Negative	M-H	1	2.86
cis-Aconitic acid	TargetCompound	C6H6O6	173.0092	Negative	M-H	1	5.58
Citric acid	TargetCompound	C6H8O7	191.0197	Negative	M-H	1	7.6
Citric acid-13C3	InternalStandard	[13]C3C3H8O7	194.0298	Negative	M-H	1	7.8
Cysteic acid	TargetCompound	C3H7NO5S	167.9972	Negative	M-H	1	8.25
Dihexose	TargetCompound	C12H22O11	387.1144	Negative	M+HCO2	1	8.4
Dihydroxybenzeneacetic acid	TargetCompound	C8H8O4	167.035	Negative	M-H	1	2.35
Dihydroxybenzoic acid	TargetCompound	C7H6O4	153.0193	Negative	M-H	1	1.66
Dimethyluric acid	TargetCompound	C7H8N4O3	195.0524	Negative	M-H	1	3.39
Ethylmalonic acid / Methylsuccinic acid / Glutaric	TargetCompound	C5H8O4	131.035	Negative	M-H	1	1.58
Fructose-13C6	InternalStandard	[13]C6H12O6	221.0529	Negative	M+Cl	1	6.77
Fumaric acid	TargetCompound	C4H4O4	115.0037	Negative	M-H	1	1.6
Galacturonic acid	TargetCompound	C5H10O5	149.0456	Negative	M-H	1	5.51
Gluconic acid	TargetCompound	C6H12O7	195.051	Negative	M-H	1	7.52
Glucuronic acid	TargetCompound	C6H10O7	193.0354	Negative	M-H	1	7.47
Glucuronolactone	TargetCompound	C6H8O6	175.0248	Negative	M-H	1	4.78
Glutamic acid-d5	InternalStandard	C5H4NO4D5	151.0773	Negative	M-H	1	8.06
Glutathione	TargetCompound	C10H17N3O6S	306.0765	Negative	M-H	1	7.83
Glycerophospho-N-palmitoyl ethanolamine	TargetCompound	C21H44NO7P	452.2783	Negative	M-H	1	5.54
Gly-Leu	TargetCompound	C8H16N2O3	187.1088	Negative	M-H	1	7.77
GMP	TargetCompound	C10H14N5O8P	362.0507	Negative	M-H	1	9.75
Guanidoacetic acid	TargetCompound	C3H7N3O2	116.0466	Negative	M-H	1	7.86
Guanosine	TargetCompound	C10H13N5O5	282.0844	Negative	M-H	1	6.5
Hexose	TargetCompound	C6H12O6	215.0328	Negative	M+Cl	1	7.2
Hexose 6-phosphate	TargetCompound	C6H13O9P	259.0224	Negative	M-H	1	9.35
Hippuric acid	TargetCompound	C9H9NO3	178.051	Negative	M-H	1	1.51
Hydroxymethylglutaric acid	TargetCompound	C6H10O5	161.0456	Negative	M-H	1	1.66
Hydroxyphenyllactic acid	TargetCompound	C9H10O4	181.0506	Negative	M-H	1	2.43
Hypoxanthine-13C5	InternalStandard	[13]C5H4N4O	140.048	Negative	M-H	1	5.2
IMP	TargetCompound	C10H13N4O8P	347.0398	Negative	M-H	1	7.72
Indolelactic acid	TargetCompound	C11H11NO3	204.0666	Negative	M-H	1	2.05
Indoxyl sulfate	TargetCompound	C8H7NO4S	212.0023	Negative	M-H	1	5.28
Lactic acid	TargetCompound	C3H6O3	89.0244	Negative	M-H	1	4.2
Maleic acid	TargetCompound	C4H4O4	115.0037	Negative	M-H	1	2.3
Malic acid	TargetCompound	C4H6O5	133.0143	Negative	M-H	1	4.66
Malonic acid	TargetCompound	C3H4O4	103.0037	Negative	M-H	1	2.97
Mandelic acid / Hydroxyphenylacetic acid	TargetCompound	C8H8O3	151.0401	Negative	M-H	1	1.62
Mannitol / Iditol	TargetCompound	C6H14O6	217.0484	Negative	M+Cl	1	7.36
Mesaconic acid / citraconic acid / itaconic acid / [illegible]	TargetCompound	C5H6O4	129.0193	Negative	M-H	1	1.61
Methoxyhydroxyphenylethyleneglycol sulfate	TargetCompound	C9H12O7S	263.0231	Negative	M-H	1	5.09
Methylpentose	TargetCompound	C6H12O5	209.0667	Negative	M+HCO2	1	5.87
Methyluric acid	TargetCompound	C6H6N4O3	181.0367	Negative	M-H	1	5.25
N-Acetylalanine	TargetCompound	C5H9NO3	130.051	Negative	M-H	1	8.01
N-Acetylaspartic acid	TargetCompound	C6H9NO5	174.0408	Negative	M-H	1	4.64

N-Acetyl-Asp-Glu	TargetCompound	C11H16N2O8	303.0834	Negative	M-H	1	5.5
N-Acetyl-D-galactosamine 4-sulphate	TargetCompound	C8H15NO9S	300.0395	Negative	M-H	1	7.26
N-Acetylglutamic acid	TargetCompound	C7H11NO5	188.0565	Negative	M-H	1	4.73
N-Acetylglutamine	TargetCompound	C7H12N2O4	187.0724	Negative	M-H	1	4.93
N-Acetylneuraminic acid	TargetCompound	C11H19NO9	308.0987	Negative	M-H	1	7.46
N-Acetyl-tryptophan	TargetCompound	C13H14N2O3	245.0932	Negative	M-H	1	1.55
Orotic acid	TargetCompound	C5H4N2O4	155.0098	Negative	M-H	1	6.12
Orotidine	TargetCompound	C10H12N2O8	287.0521	Negative	M-H	1	7.79
Oxoproline	TargetCompound	C5H7NO3	128.0353	Negative	M-H	1	2.75
P-Cresol	TargetCompound	C7H8O	107.0502	Negative	M-H	1	1.62
p-Cresol sulfate	TargetCompound	C7H8O4S	187.0071	Negative	M-H	1	1.62
Pentose	TargetCompound	C5H10O5	149.0456	Negative	M-H	1	5.51
Pseudouridine	TargetCompound	C9H12N2O6	243.0623	Negative	M-H	1	6.2
Quinic acid	TargetCompound	C7H12O6	191.0561	Negative	M-H	1	5.17
Ribose 5-phosphate	TargetCompound	C5H11O8P	229.0119	Negative	M-H	1	9.2
Saccharopine	TargetCompound	C11H20N2O6	275.1249	Negative	M-H	1	10.03
S-adenosylhomocysteine	TargetCompound	C14H20N6O5S	383.1143	Negative	M-H	1	9.42
Salicyluric acid	TargetCompound	C9H9NO4	194.0459	Negative	M-H	1	4.15
ST 19:2;O2;S	TargetCompound	C19H28O5S	367.1585	Negative	M-H	1	1.64
Succinic acid	TargetCompound	C4H6O4	117.0193	Negative	M-H	1	1.66
Succinic acid-d4	InternalStandard	C4H2O4D4	121.0444	Negative	M-H	1	1.62
Threonic acid	TargetCompound	C4H8O5	135.0299	Negative	M-H	1	7.5
Trimethyluric acid	TargetCompound	C8H10N4O3	209.068	Negative	M-H	1	2.5
UMP	TargetCompound	C9H13N2O9P	323.0286	Negative	M-H	1	8.35
Ureidopropionic acid	TargetCompound	C4H8N2O3	131.0462	Negative	M-H	1	4.66
Uric acid	TargetCompound	C5H4N4O3	167.0211	Negative	M-H	1	6.56
Uridine	TargetCompound	C9H12N2O6	243.0623	Negative	M-H	1	5.17
Vitamin C	TargetCompound	C6H8O6	175.0248	Negative	M-H	1	4.8
Xanthosine	TargetCompound	C10H12N4O6	283.0684	Negative	M-H	1	5.99
Xanthurenic acid	TargetCompound	C10H7NO4	204.0302	Negative	M-H	1	5.37
Xylitol / Arabitol	TargetCompound	C5H12O5	151.0612	Negative	M-H	1	6.58

Suppl. Table 4. Islet donors

Condition	Sex	HbA1c (%)	Fasting glucose (mmol/L)
Healthy	F	5.3	6.47
Healthy	M	5.1	5.39
Healthy	M	5.6	6.07
Healthy	F	6	5.06
Healthy	F	5.6	4.95
Healthy	M	5.3	5.9
Healthy	M	5.1	4.7
Healthy	M	5.1	-
Healthy	M	5.1	-
T2D	M	7.3	10.96
T2D	M	7.6	10.65
T2D	F	7.3	9.73
T2D	F	7.6	9.87
T2D	F	8.7	12.17
T2D	M	7.1	10.63
T2D	M	8	10.17
T2D	F	7.8	11.81
T2D	M	8	12.96

Suppl. Table 5. Primer sequences used for RT-PCR

Gene	Forward (5′–3′)	Reverse (3′–5′)
Mouse *Il6*	CGATGATGCACTTGCAGAAA	ACTCCAGAAGACCAGAGGAA
Mouse *Il6ra*	GCCACCGTTACCCTGATTTG	TCCTGTGGTAGTCCATTCTCTG
Mouse *Ffar4*	CGATTTGCACATTGGATTGGC	AGTATGCCAAGCTCAGCGTA
Mouse *Ins1*	CAGAGAGGAGGTACTTTGGACTATAAA	GCCATGTTGAAACAATGACCT
Mouse *Gcg*	TTCCTTTGCTGCCTGGCCCT	TTCCCAGACAGAAGCGCATGAG
Mouse *Sst*	CCACCGGGAAACAGGAAC	GCTCCAGCCTCATCTCGTC
Mouse *Gapdh*	CCATTTTGTCTACGGGACGA	GGGTTCCTATAAATACGGACTGC

Gating strategy of islet macrophages
(related to Figure 5e-g, and k; Suppl. Figure 5b and c)

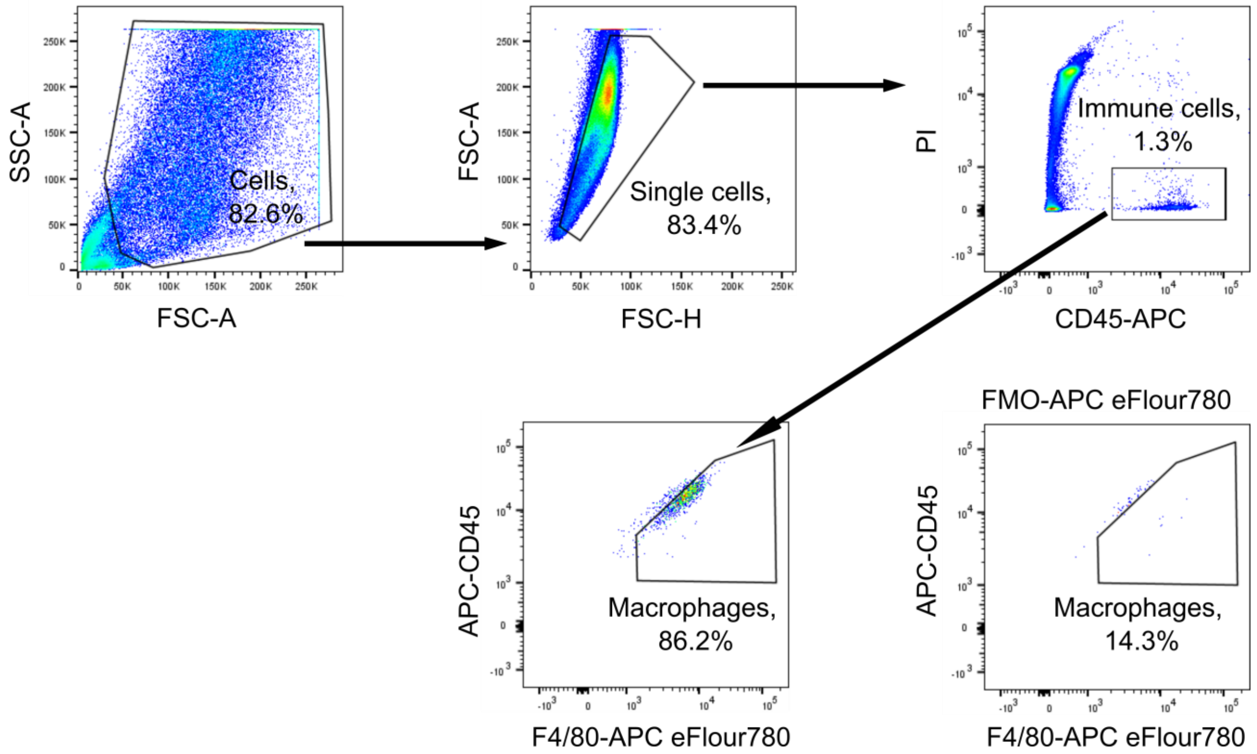

Gating strategy of epithelial cells
(related to Suppl. Figure 2o and r)

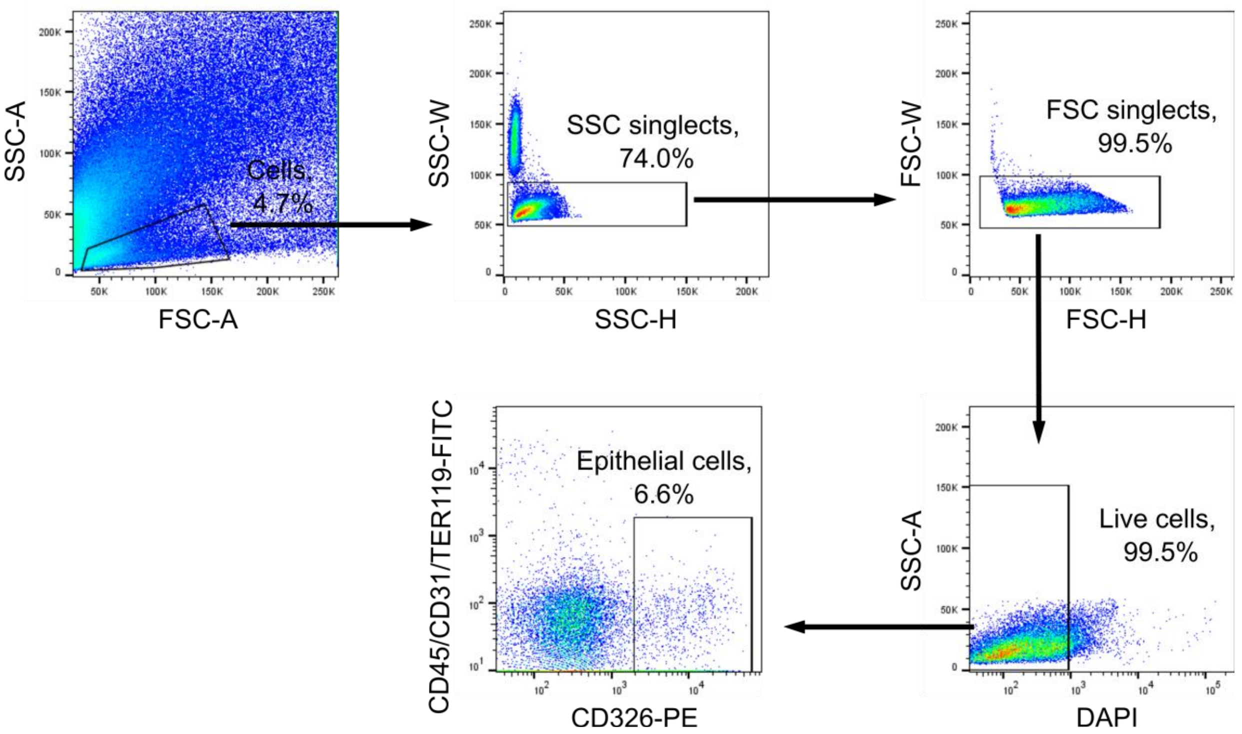

Uncropped gel picture

Suppl. Figure 1b

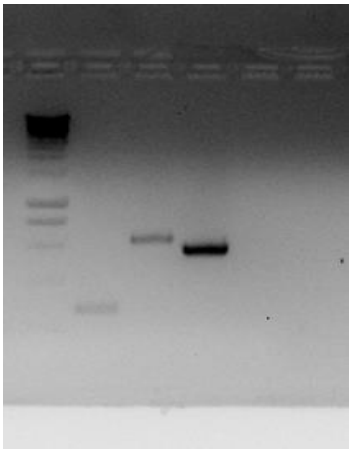

Supplement: Supplementary file 1 — Supplementary Information [file 41467_2025_58706_MOESM1_ESM.pdf]
