# Supplementary material for: FFAR4-mediated IL-6 release from islet macrophages promotes insulin secretion and is compromised in type-2 diabetes
Source: Nat Commun. 2025 Apr 10;16:3422. doi: 10.1038/s41467-025-58706-5 (PMC11986018; doi:10.1038/s41467-025-58706-5)
Supplement: Supplementary file 2 — Reporting Summary [file 41467_2025_58706_MOESM2_ESM.pdf]

nature portfolio

Corresponding author(s): Stefan Offermanns

Last updated by author(s): Mar 17, 2025

# Reporting Summary

Nature Portfolio wishes to improve the reproducibility of the work that we publish. This form provides structure for consistency and transparency in reporting. For further information on Nature Portfolio policies, see our Editorial Policies and the Editorial Policy Checklist.

## Statistics

For all statistical analyses, confirm that the following items are present in the figure legend, table legend, main text, or Methods section.

| n/a | Confirmed | |
|---|---|---|
| ☐ | ☒ | The exact sample size (*n*) for each experimental group/condition, given as a discrete number and unit of measurement |
| ☐ | ☒ | A statement on whether measurements were taken from distinct samples or whether the same sample was measured repeatedly |
| ☐ | ☒ | The statistical test(s) used AND whether they are one- or two-sided<br>*Only common tests should be described solely by name; describe more complex techniques in the Methods section.* |
| ☒ | ☐ | A description of all covariates tested |
| ☐ | ☒ | A description of any assumptions or corrections, such as tests of normality and adjustment for multiple comparisons |
| ☐ | ☒ | A full description of the statistical parameters including central tendency (e.g. means) or other basic estimates (e.g. regression coefficient) AND variation (e.g. standard deviation) or associated estimates of uncertainty (e.g. confidence intervals) |
| ☐ | ☒ | For null hypothesis testing, the test statistic (e.g. *F*, *t*, *r*) with confidence intervals, effect sizes, degrees of freedom and *P* value noted<br>*Give P values as exact values whenever suitable.* |
| ☒ | ☐ | For Bayesian analysis, information on the choice of priors and Markov chain Monte Carlo settings |
| ☒ | ☐ | For hierarchical and complex designs, identification of the appropriate level for tests and full reporting of outcomes |
| ☒ | ☐ | Estimates of effect sizes (e.g. Cohen's *d*, Pearson's *r*), indicating how they were calculated |

*Our web collection on statistics for biologists contains articles on many of the points above.*

## Software and code

Policy information about availability of computer code

| | |
|---|---|
| Data collection | qRT-PCR: LightCycler 480 software 1.5.0SP3 |
| Data analysis | statistics and graphs: GraphPad Prism v10.1.2<br>FACS analysis:FlowJo v10.6.2<br>image analysis: ImageJ/FIJI v1.0, Leica LAS-AF Lite v3.3.10134.0, Inkscape v1.3.2 |

For manuscripts utilizing custom algorithms or software that are central to the research but not yet described in published literature, software must be made available to editors and reviewers. We strongly encourage code deposition in a community repository (e.g. GitHub). See the Nature Portfolio guidelines for submitting code & software for further information.

## Data

Policy information about availability of data

All manuscripts must include a data availability statement. This statement should provide the following information, where applicable:

- Accession codes, unique identifiers, or web links for publicly available datasets
- A description of any restrictions on data availability
- For clinical datasets or third party data, please ensure that the statement adheres to our policy

The lipidomics and metabolomics data generated in this study have been deposited to MetaboLights repository with the study identifier MTBLS12317. The source data underlying Figs. 1a-f, 2a-m, 3a-h, 4a-g, 5a-k, 6a-c, and Extended data figure 1c-g, 2a-r, 3b, 4a-b, 5a-e, 6a-b are provided as Source data file.

# Research involving human participants, their data, or biological material

Policy information about studies with human participants or human data. See also policy information about sex, gender (identity/presentation), and sexual orientation and race, ethnicity and racism.

| | |
|---|---|
| Reporting on sex and gender | No |
| Reporting on race, ethnicity, or other socially relevant groupings | Due to limited sample size, race, ethnicity, or other socially relevant groupings were not considered in the study design. |
| Population characteristics | All patients were Chinese and diagnosed with or without type 2 diabetes. |
| Recruitment | Written informed consent was obtained from all subjects before their participation. Human pancreatic samples were obtained from patients undergoing dissecting pancreatic surgery in the Pancreas Center, The First Affiliated Hospital of Xi'an Jiaotong University. Patients identities were not known to the researchers except for information of gender and age. |
| Ethics oversight | The work on human pancreatic samples was approved by the ethical committee of Xi'an Jiaotong University (XJTU1AF2024LSYY-308) and conforms to the guidelines of the 2000 Helsinki declaration. Written informed consent was obtained from all subjects before their participation. |

Note that full information on the approval of the study protocol must also be provided in the manuscript.

# Field-specific reporting

Please select the one below that is the best fit for your research. If you are not sure, read the appropriate sections before making your selection.

☒ Life sciences ☐ Behavioural & social sciences ☐ Ecological, evolutionary & environmental sciences

For a reference copy of the document with all sections, see nature.com/documents/nr-reporting-summary-flat.pdf

# Life sciences study design

All studies must disclose on these points even when the disclosure is negative.

| | |
|---|---|
| Sample size | Sample size was determined on basis of trial experiments or experiments done previously. |
| Data exclusions | Samples were excluded in cases where cDNA quality or tissue quality after processing was poor (below commonly accepted standards). Animals were excluded from experiments if they showed any signs of sickness (weight loss more than 20 %, skin infection, shaggy fur, loss of / or reduced movements, abnormal breathing). |
| Replication | Number of independent experiments are given in the Figure legends. Each experiment was repeated at least twice under independent conditions. |
| Randomization | No randomization was used for samples. In animal experiments, mice were caged with blinded cage numbers and random orders. |
| Blinding | The investigator was blinded to the group allocation and during the experiment. In animal experiments, mice were caged with blinded cage numbers and random orders. |

# Reporting for specific materials, systems and methods

We require information from authors about some types of materials, experimental systems and methods used in many studies. Here, indicate whether each material, system or method listed is relevant to your study. If you are not sure if a list item applies to your research, read the appropriate section before selecting a response.

## Materials & experimental systems

| n/a | Involved in the study | |
|---|---|---|
| ☐ | ☒ | Antibodies |
| ☒ | ☐ | Eukaryotic cell lines |
| ☒ | ☐ | Palaeontology and archaeology |
| ☐ | ☒ | Animals and other organisms |
| ☒ | ☐ | Clinical data |
| ☒ | ☐ | Dual use research of concern |
| ☒ | ☐ | Plants |

## Methods

| n/a | Involved in the study | |
|---|---|---|
| ☒ | ☐ | ChIP-seq |
| ☐ | ☒ | Flow cytometry |
| ☒ | ☐ | MRI-based neuroimaging |

## Antibodies

| | |
|---|---|
| Antibodies used | Rabbit anti-IL-6 (Invitrogen, #P620), rabbit anti-Glucagon (Cell Signaling, #2760S), rabbit anti-Somatostatin (Santa Cruz, #sc-13099), guinea pig anti-Insulin (GeneTex, #GTX27842), rat anti-F4/80 (Bio-Rad, #MCA497R), rat anti-CD45, APC (Invitrogen, #17-0451-82), rat anti-CD45, FITC (BD Bioscience, #553079) rat anti-F4/80, APC-eFluorTM 780 (Invitrogen #47-4801-82), rat anti-F4/80, PE (BioLegend, #123110), rat anti-CD31, FITC (BD, #558738), rat anti-TER-119, FITC (Invitrogen, #11-5921-82), rat anti-CD326, PE (BioLegend, #118205), donkey anti-rat secondary antibody, Alexa FluorTM 488 (Invitrogen, #A-21208), donkey anti-rat secondary antibody, Alexa FluorTM 594 (Invitrogen, #A-21209), donkey anti-rabbit secondary antibody, Alexa FluorTM 594 (Invitrogen, #A-21207), goat anti-rabbit secondary antibody, Alexa FluorTM 647 (Invitrogen, #A-21244) |
| Validation | All antibodies were validated according to instruction on the manufactures' website. |

## Animals and other research organisms

Policy information about studies involving animals; ARRIVE guidelines recommended for reporting animal research, and Sex and Gender in Research

| | |
|---|---|
| Laboratory animals | Stated in Method. C57BL/6 background, Experiments were performed with littermates as controls. Male and female animals (8-20 weeks of age) were used. Mice were housed under a 12-hour light-dark cycle with free access to food and water and under specific pathogen-free conditions. |
| Wild animals | Not involved |
| Reporting on sex | Sex was not considered of relevance in the study design as we describe a general mechanism that is likely not to be sex-specific. Therefore, only male animals were used in this study to keep the total number of animals used in experiments as low as possible. |
| Field-collected samples | Not invovled |
| Ethics oversight | Animal experiments were approved by the Institutional Animal Care and Use Committee Regierungspräsidium Darmstadt Germany and the Ethical Committee of Xi'an Jiaotong University, China |

Note that full information on the approval of the study protocol must also be provided in the manuscript.

## Plants

| | |
|---|---|
| Seed stocks | *Report on the source of all seed stocks or other plant material used. If applicable, state the seed stock centre and catalogue number. If plant specimens were collected from the field, describe the collection location, date and sampling procedures.* |
| Novel plant genotypes | *Describe the methods by which all novel plant genotypes were produced. This includes those generated by transgenic approaches, gene editing, chemical/radiation-based mutagenesis and hybridization. For transgenic lines, describe the transformation method, the number of independent lines analyzed and the generation upon which experiments were performed. For gene-edited lines, describe the editor used, the endogenous sequence targeted for editing, the targeting guide RNA sequence (if applicable) and how the editor was applied.* |
| Authentication | *Describe any authentication procedures for each seed stock used or novel genotype generated. Describe any experiments used to assess the effect of a mutation and, where applicable, how potential secondary effects (e.g. second site T-DNA insertions, mosiacism, off-target gene editing) were examined.* |

## Flow Cytometry

### Plots

Confirm that:

☒ The axis labels state the marker and fluorochrome used (e.g. CD4-FITC).

☒ The axis scales are clearly visible. Include numbers along axes only for bottom left plot of group (a 'group' is an analysis of identical markers).

☒ All plots are contour plots with outliers or pseudocolor plots.

☒ A numerical value for number of cells or percentage (with statistics) is provided.

### Methodology

| | |
|---|---|
| Sample preparation | Stated in Method. Islets and intestinal epithelial cells were harvested and stained with antibodies as stated in the Method part under Cell sorting and flow cytometry analysis. |
| Instrument | FACSMelody cell sorter, FACSAria III cell sorter, FACSCanto II flow cytometry system, LSRFortessa cell analyzer |
| Software | BD FACSDiva v6.1 |

| | |
|---|---|
| Cell population abundance | N/A. |
| Gating strategy | The gating strategies are provided in Extended Data Fig. 3b, 4a-b, 6a-b. |

☒ Tick this box to confirm that a figure exemplifying the gating strategy is provided in the Supplementary Information.
